# Identifying Replicable Subgroups in Neurodevelopmental Conditions Using Resting-State Functional Magnetic Resonance Imaging Data

**DOI:** 10.1001/jamanetworkopen.2023.2066

**Published:** 2023-03-13

**Authors:** Marlee M. Vandewouw, Jessica Brian, Jennifer Crosbie, Russell J. Schachar, Alana Iaboni, Stelios Georgiades, Robert Nicolson, Elizabeth Kelley, Muhammad Ayub, Jessica Jones, Margot J. Taylor, Jason P. Lerch, Evdokia Anagnostou, Azadeh Kushki

**Affiliations:** 1 Autism Research Centre, Bloorview Research Institute, Holland Bloorview Kids Rehabilitation Hospital, Toronto, Ontario, Canada; 2 Institute of Biomedical Engineering, University of Toronto, Toronto, Ontario, Canada; 3 Department of Paediatrics, University of Toronto, Toronto, Ontario, Canada; 4 Department of Psychiatry, University of Toronto, Toronto, Ontario, Canada; 5 Department of Psychiatry, The Hospital for Sick Children, Toronto, Ontario, Canada; 6 Department of Psychiatry and Behavioural Neurosciences, McMaster University, Hamilton, Ontario, Canada; 7 Department of Psychiatry, Western University, London, Ontario, Canada; 8 Department of Psychology, Queen’s University, Kingston, Ontario, Canada; 9 Centre for Neuroscience Studies, Queen’s University, Kingston, Ontario, Canada; 10 Department of Psychiatry, Queen’s University, Kingston, Ontario, Canada; 11 Program in Neurosciences & Mental Health, The Hospital for Sick Children, Toronto, Ontario, Canada; 12 Department of Diagnostic Imaging, The Hospital for Sick Children, Toronto, Ontario, Canada; 13 Department of Psychology, University of Toronto, Toronto, Ontario, Canada; 14 Department of Medical Imaging, University of Toronto, Toronto, Ontario, Canada; 15 Wellcome Centre for Integrative Neuroimaging, FMRIB, Nuffield Department of Clinical Neurosciences, University of Oxford, Oxford, United Kingdom; 16 Department of Medical Biophysics, University of Toronto, Toronto, Ontario, Canada

## Abstract

**Importance:**

Neurodevelopmental conditions, such as autism spectrum disorder (ASD), attention-deficit/hyperactivity disorder (ADHD), and obsessive-compulsive disorder (OCD), have highly heterogeneous and overlapping phenotypes and neurobiology. Data-driven approaches are beginning to identify homogeneous transdiagnostic subgroups of children; however, findings have yet to be replicated in independently collected data sets, a necessity for translation into clinical settings.

**Objective:**

To identify subgroups of children with and without neurodevelopmental conditions with shared functional brain characteristics using data from 2 large, independent data sets.

**Design, Setting, and Participants:**

This case-control study used data from the Province of Ontario Neurodevelopmental (POND) network (study recruitment began June 2012 and is ongoing; data were extracted April 2021) and the Healthy Brain Network (HBN; study recruitment began May 2015 and is ongoing; data were extracted November 2020). POND and HBN data are collected from institutions across Ontario and New York, respectively. Participants who had diagnoses of ASD, ADHD, and OCD or were typically developing (TD); were aged between 5 and 19 years; and successfully completed the resting-state and anatomical neuroimaging protocol were included in the current study.

**Main Outcomes and Measures:**

The analyses consisted of a data-driven clustering procedure on measures derived from each participant’s resting-state functional connectome, performed independently on each data set. Differences between each pair of leaves in the resulting clustering decision trees in the demographic and clinical characteristics were tested.

**Results:**

Overall, 551 children and adolescents were included from each data set. POND included 164 participants with ADHD; 217 with ASD; 60 with OCD; and 110 with TD (median [IQR] age, 11.87 [9.51-14.76] years; 393 [71.2%] male participants; 20 [3.6%] Black, 28 [5.1%] Latino, and 299 [54.2%] White participants) and HBN included 374 participants with ADHD; 66 with ASD; 11 with OCD; and 100 with TD (median [IQR] age, 11.50 [9.22-14.20] years; 390 [70.8%] male participants; 82 [14.9%] Black, 57 [10.3%] Hispanic, and 257 [46.6%] White participants). In both data sets, subgroups with similar biology that differed significantly in intelligence as well as hyperactivity and impulsivity problems were identified, yet these groups showed no consistent alignment with current diagnostic categories. For example, there was a significant difference in Strengths and Weaknesses ADHD Symptoms and Normal Behavior Hyperactivity/Impulsivity subscale (SWAN-HI) between 2 subgroups in the POND data (C and D), with subgroup D having increased hyperactivity and impulsivity traits compared with subgroup C (median [IQR], 2.50 [0.00-7.00] vs 1.00 [0.00-5.00]; *U* = 1.19 × 10^4^; *P* = .01; η^2^ = 0.02). A significant difference in SWAN-HI scores between subgroups g and d in the HBN data was also observed (median [IQR], 1.00 [0.00-4.00] vs 0.00 [0.00-2.00]; corrected *P* = .02). There were no differences in the proportion of each diagnosis between the subgroups in either data set.

**Conclusions and Relevance:**

The findings of this study suggest that homogeneity in the neurobiology of neurodevelopmental conditions transcends diagnostic boundaries and is instead associated with behavioral characteristics. This work takes an important step toward translating neurobiological subgroups into clinical settings by being the first to replicate our findings in independently collected data sets.

## Key Points

**Question:**

Are subgroups derived from measures of brain function among children and adolescents with neurodevelopmental conditions replicable across independently collected data sets?

**Findings:**

In this case-control study using resting-state data from 2 network data sets with a total of 1102 individuals aged 5 to 19 years with and without neurodevelopmental conditions, subgroups with similar biology that differed significantly in intelligence as well as hyperactivity and impulsivity problems were identified. However, these groups showed no consistent alignment with diagnostic categories.

**Meaning:**

In this study, homogeneity in the neurobiology of neurodevelopmental conditions corresponded to behavior, not diagnostic category; these findings are replicable in independent cohorts, taking an important step toward translating neurobiological subgroups into clinical settings.

## Teaser

This case-control study identifies subgroups of children with and without neurodevelopmental conditions with shared brain characteristics using functional magnetic resonance imaging (fMRI) data from 2 large, independent data sets.

## Introduction

Autism spectrum disorder (ASD), attention-deficit/hyperactivity disorder (ADHD), and obsessive-compulsive disorder (OCD) are neurodevelopmental conditions clinically defined based on distinct behavioral criteria.^1^ However, an increasing body of evidence suggests that these conditions are highly heterogeneous in biology and phenotype within each condition^2,3,4^ and significantly overlapping.^5,6,7,8,9,10,11,12^ These observations pose significant challenges to traditional case-control studies, especially with relatively small sample sizes,^13^ and have resulted in discrepant findings across various studies. For example, investigations attempting to characterize the functional connectome in individuals with ASD, ADHD, and OCD compared with typically developing (TD) populations are highly mixed.^14,15,16^ The seemingly contradictory findings reflect the heterogeneous nature of these conditions^17,18^ as well as suggest that differences in the topography of the functional connectome, such as integration and segregation between resting state networks, may better explain connectivity patterns in neurodevelopmental conditions rather than the strength of individual connections.^19,20,21,22,23^ These topographical differences have frequently been described in all 3 conditions,^24,25,26^ and there is increasing evidence that these differences are shared across conditions.^9,10^

To disentangle these findings, a shift from traditional case-control designs to data-driven approaches, which transcend diagnostic boundaries to identify groups that are homogeneous in their neurobiology, is necessary. An emerging body of literature using data-driven approaches supports the idea that the diagnostic categories of ASD, ADHD, and OCD are not associated with unique underlying neurobiological mechanisms^27,28^ and often do not predict treatment outcome.^29^ This motivates the need for the discovery of homogeneous groups that can accelerate the development of targeted and personalized treatment approaches, interventions, supports, and accommodations that fit the diverse profiles of strengths and needs of children with neurodevelopmental conditions.

To this end, several studies have used measures of brain function or structure to identify transdiagnostic subgroups of neurodevelopmental conditions, consistently demonstrating a misalignment between data-driven groupings and existing diagnostic categories.^9,10,12,27,28,30,31,32^ The first contribution of this article is to characterize the heterogeneity across neurodevelopmental conditions by identifying cross-diagnosis subgroups of children and adolescents with and without neurodevelopmental conditions using measures of integration and segregation of the functional connectome.

Despite the promise of data-driven approaches and the encouraging preliminary reports, the replicability and generalizability of these findings remains an open question in the field^33^ and a critical gap to clinical translation of the findings.^13,34^ To date, this issue has been addressed partly by using subsampling within a data set to enhance generalizability^27,28^; however, to our knowledge, subgroupings within neurodevelopmental conditions based on neuroimaging data have not been replicated across independently collected data sets. The second contribution of this article is to be the first, to our knowledge, to address this replication gap by examining subgroups across 2 large, independently collected, cross-condition data sets, namely the Province of Ontario Neurodevelopmental Disorders (POND) network and the Healthy Brains Network (HBN).

## Methods

Both the POND network and HBN studies were approved by the appropriate research ethics boards, and the current study was approved by the Holland Bloorview Kids Rehabilitation Hospital’s research ethics board; written informed consent and/or verbal assent was obtained from the primary caregivers and/or participants (eMethods in Supplement 1). This study followed the Strengthening the Reporting of Observational Studies in Epidemiology (STROBE) reporting guideline for case-control studies.

### Participants

For the primary cohort, participants were drawn from the POND Network data set (exported April 2021)^35^, and data from the Healthy Brain Network^36,37^ was used as the replication cohort (exported November 2020) (eMethods in Supplement 1). Overall, 717 POND participants (210 with ADHD; 300, ASD; 69, OCD; and 138, TD) and 966 HBN participants (672, ADHD; 111, ASD; 12, OCD; and 171, TD) aged between 5 and 19 years were included in the current study based on successful completion of the resting-state and anatomical imaging protocols and presence of phenotypic data. Details on phenotypic measures used to characterize the POND and HBN samples are provided in the eMethods in Supplement 1.

Both datasets used self- or parent-reported race and ethnicity, per the protocols of the larger POND and HBN studies. In the POND data set, racial groups were defined according to the Canadian Institute for Health Information standards and included Black, East Asian, Indigenous, Latino, Middle Eastern, other, South Asian, Southeast Asian, and White. Participants were classified into multiple categories if they were of mixed race; those who did not identify as one of the groups were categorized as other. In the HBN data set, categories were defined according to US Census guidelines and included American Indian or Alaskan Native, Asian, Black, Hispanic, 2 or more races, Native Hawaiian or other Pacific Islander, other, and White. Participants of mixed race were classified as such, and thus participants were only assigned to 1 category; those who did not identify as any of the census groups were categorized as other. Due to low sample size, categories for both datasets were collapsed into minoritized racial and ethnic group and White for statistical tests.

### Neuroimaging Data

Five minutes of resting-state data and anatomical brain images were collected as part of the POND and HBN studies and preprocessed. Propensity scores were used to match the POND and HBN participants who passed quality control on age, sex, and motion. Full details on data acquisition, preprocessing, and propensity score matching can be found in eMethods and eTable 1 in Supplement 1.

### Connectome Construction

Connectome nodes were defined using the cortical atlas from Schaefer et al,^38^ supplemented by the Melbourne subcortical atlas,^39^ as this parcellation scheme is highly representative across alternative connectome construction pipelines,^40^ resulting in 232 nodes. The parcels were categorized into 8 functional networks: visual, somatomotor, dorsal attention, ventral attention and salience, limbic, frontoparietal control, default mode, and subcortical (eFigure 1 in Supplement 1). Pairwise Pearson correlations between parcel-averaged preprocessed time series were computed as the edge weights between pairs of nodes. The edge weights were harmonized to account for acquisition site effects across both data sets, and the influence of scanner, age, and sex were removed (eMethods in Supplement 1). The connectomes were thresholded to remove spurious connections and produce more biologically plausible connectomes.^40,41^ For each participant, nodal measures of integration (betweenness centrality^42,43^) and segregation (clustering coefficient^42^) were extracted and *z* scored.

### Clustering

**Figure 1.**
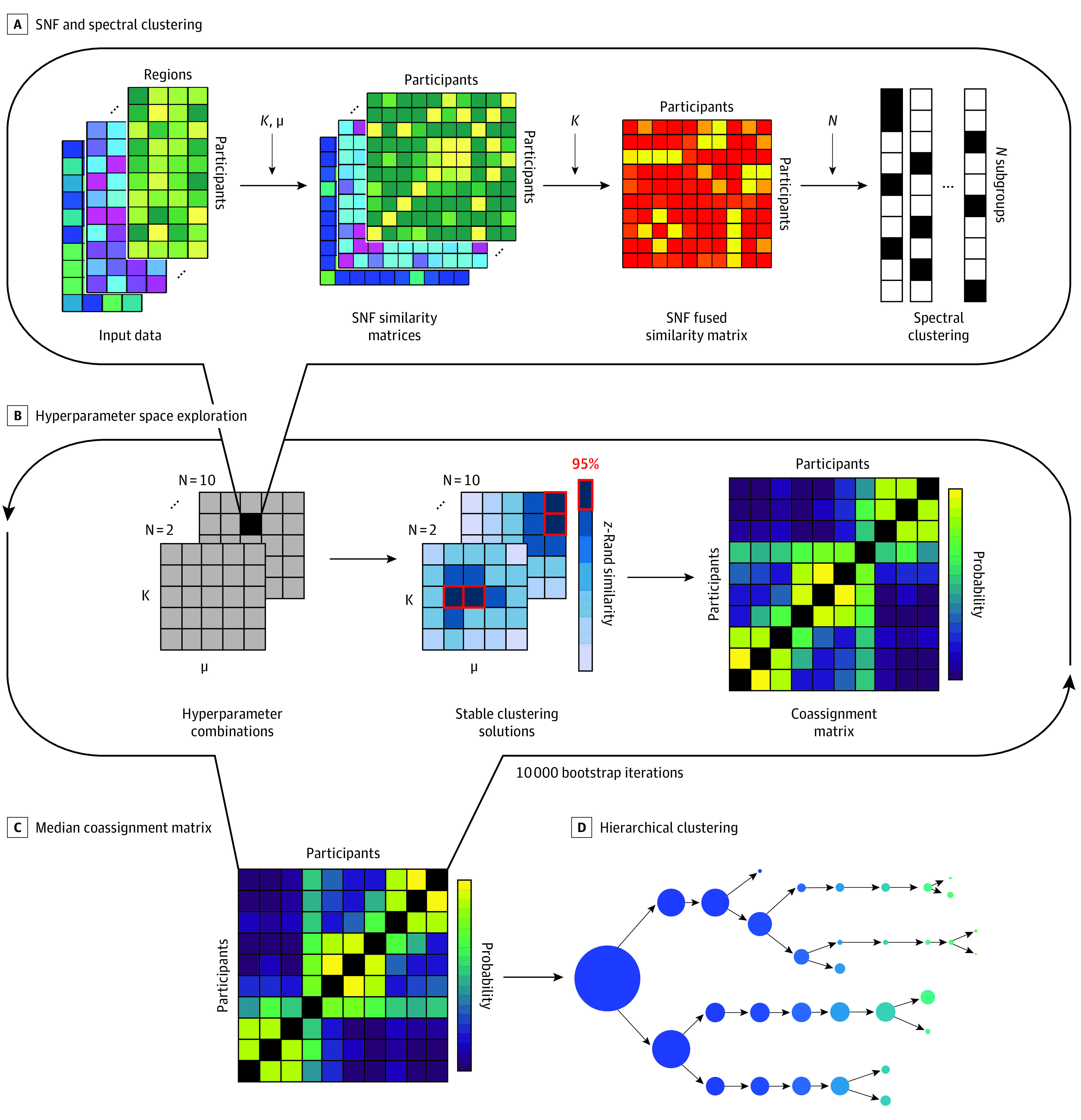
Clustering Pipeline A, The similarity network fusion (SNF) pipeline was used to construct similarity matrices for each network-measure pair, which were subsequently fused and clustered. B, This procedure was repeated over a wide range of SNF hyperparameters (*K* and μ), each time for a prespecified number of clusters, *N*, ranging from 2 to 10. Stable solutions were identified using the z-Rand similarity index and used to construct a participant coassignment matrix. C, This procedure was repeated for 10 000 subsamples of participants, taking the median across all coassignment matrices. D, Hierarchical clustering was used to identify the emergence of 2 to 10 clusters from the final coassignment matrix.

Clustering was performed separately on the POND and HBN data sets, and the pipeline is presented in Figure 1. In the first section (Figure 1A), similarity network fusion (SNF^44^) was used to compute similarity matrices for each measure-network pair (eg, segregation of the visual network) using the Euclidean distance across all nodal measures belonging to the network and SNF’s *K*-nearest neighbors weighted similarity kernel. The 16 similarity matrices (8 networks × 2 measures) were then fused using SNF, from which spectral clustering can be used to identify a prespecified number of subgroups.

Due to SNF’s dependence on 2 free hyperparameters (μ and *K*), 10 000 clustering solutions were obtained using different combinations of hyperparameters (Figure 1B and eMethods in Supplement 1). A participant coassignment matrix was then generated by computing the percentage of times 2 participants were clustered in the same subgroup across cluster solutions that were stable across the hyperparameter space.

The clustering procedure was performed on 10 000 subsampling iterations to increase robustness of the final clustering solution, selecting 63.2% of the sample in each iteration; a final coassignment matrix was constructed by computing the median across all subsampling iterations (Figure 1C). Hierarchical clustering was performed on the final coassignment matrix (Figure 1D) to identify subgroups across the full range of number of clusters (2-10). Hierarchical clustering constructs a rooted tree, or a dendrogram, consisting of layers of nodes: the first layer contains a single cluster, representing the trivial solution, and the *n*th layer contains *n* clusters, representing the *n*th-cluster solution^45^; in each layer, a root cluster is split into 2 leaf clusters. The optimal number of clusters was identified using the Calinski-Harabasz index.^46^

### Statistical Analysis

Differences in the demographic and behavioral measures were compared among the diagnostic groups within each data set as well as compared between data sets. For the continuous measures, Kruskal-Wallis tests or 1-way analysis of variance were used, depending on normality (eTable 2 in Supplement 1); for significant omnibus tests (*P* < .05), post hoc testing was carried out using the Dunn procedure with Bonferroni-corrected *P* values (corrected *P*_ _< .05). For nominal categorical variables (sex and acquisition scanner), χ^2^ tests were performed with post hoc pairwise *z* tests of independent proportions (corrected *P* < .05). For ordinal categorical variables (socioeconomic variables in the POND data set), ordinal regression was performed, testing for all pairwise between-group differences (corrected *P* < .05).

To determine which brain measures were associated with the split from a root cluster into its 2 leaf clusters in each layer, we tested for a difference in means between each pair of leaf clusters in their network-averaged measures of segregation and integration. Given that we are using the same data to both define the groups via clustering and perform downstream testing, traditional statistical tests such as Mann-Whitney *U* and *t* tests would lead to inflated type I errors, as they only control for such error rates when groups are defined a priori.^47^ Thus, we used the clusterpval^48^ package in R version 4.2.1 (R Project for Statistical Computing) to produce test statistics and *P* values that are corrected for double-use of the data; the generic implementation was used, which approximates the corrected *P* values using Monte Carlo sampling, and the resulting *P* values were corrected for multiple comparisons (corrected *P* < .05). Mann-Whitney *U* or *t* tests and χ^2^ tests were used to determine differences between leaves in the demographic and behavioral measures.

For all significant tests, effect sizes were reported. For continuous measures, eta-squared (η^2^) effect sizes were used, using the ranked data for nonnormally distributed data. For categorical variables, Cramer *V* effect sizes were reported, while for ordinal variables, pseudo-*R*^2^ values were reported.^49^

## Results

### Sample Characteristics

The final data set included 551 POND participants (164 with ADHD; 217, ASD; 60, OCD; 110, TD; median [IQR] age. 11.87 [9.51-14.76] years; 393 [71.2%] male participants; 20 [3.6%] Black, 28 [5.1%] Latino, and 299 [54.2%] White participants) and 551 HBN participants (374 with ADHD; 66, ASD; 11, OCD; 100, TD; median [IQR] age, 11.50 [9.22-14.20] years; 390 [70.8%] male participants; 82 [14.9%] Black, 57 [10.3%] Hispanic, and 257 [46.6%] White participants). The final sample was reached by performing propensity matching on the individuals who passed quality control (592 POND and 756 HBN individuals) to match the data sets on age, sex, and head motion. Descriptive statistics of the POND and HBN sample characteristics are provided in Tables 1 and 2, respectively. Complete race and ethnicity data for each data set are presented in eTable 3 in Supplement 1.

**Table 1.** Descriptive Statistics of the Participant Demographics and Clinical Behavioral Measures for the Province of Ontario Neurodevelopmental Network Data Set, With the Corresponding Statistics Identifying Significant Differences Among the Diagnostic Groups

Measure^a^	Median (IQR)				Statistics			
	ADHD (n = 164)	ASD (n = 217)	OCD (n = 60)	TD (n = 110)	Test statistic^b^	*P* value	Effect size^c^	Significant post hoc^d^
Age, y	11.34 (9.47 to 13.77)	11.90 (9.51 to 15.14)	12.98 (11.32 to 15.12)	11.6 (8.94 to 13.35)	9.50	.02	0.02	ADHD < OCD
Sex, No. (%)								
Male	125 (76.2)	166 (76.5)	38 (63.3)	64 (58.2)	15.9	1.18 × 10^−3^	0.17	Female: TD, OCD > ADHD, ASD
Female	39 (23.8)	51 (23.5)	22 (36.7)	46 (41.8)				
Primary caregiver education, No. (%)^e^								
1	1 (0.6)	2 (0.9)		2 (1.8)	8.78	.03	0.03	ASD < OCD, TD
2	10 (6.1)	23 (10.6)		10 (9.1)				
3	20 (12.2)	34 (15.7)	2 (3.3)	17 (15.5)				
4	25 (15.2)	41 (18.9)	5 (8.3)	44 (40)				
5	18 (11)	31 (14.3)	5 (8.3)	32 (29.1)				
Household income, No. (%)^f^								
Low	17 (10.4)	29 (13.4)	1 (1.7)	13 (11.8)	11.76	.01	0.04	ADHD, ASD < TD
Medium	36 (22)	60 (27.6)	4 (6.7)	40 (36.4)				
High	19 (11.6)	25 (11.5)	3 (5)	39 (35.5)				
Race and ethnicity, No. (%)								
Minoritized race and ethnicity^g^	48 (29.3)	55 (25.3)	15 (25)	40 (36.4)	0.3	.96	0.03	NA
White	65 (39.6)	84 (38.7)	20 (33.3)	59 (53.6)				
Scanner, No. (%)								
SK-TT	41 (25)	90 (41.5)	37 (61.7)	16 (14.5)	81.3	1.97 × 10^−15^	0.27	SK-TT: OCD > ASD > ADHD, TD; QU-TT: ADHD, TD > ASD
QU-TT	38 (23.2)	11 (5.1)	0 (0)	31 (28.2)				
SK-PF	85 (51.8)	116 (53.5)	23 (38.3)	63 (57.3)				
Head motion, mm	11.34 (9.47 to 13.77)	11.90 (9.51 to 15.14)	12.98 (11.32 to 15.12)	11.6 (8.94 to 13.35)	13.31	4.01 × 10^−3^	0.02	ASD > OCD, TD
FSIQ	11.34 (9.47 to 13.77)	11.90 (9.51 to 15.14)	12.98 (11.32 to 15.12)	11.6 (8.94 to 13.35)	79.01	5.01 × 10^−17^	0.16	ASD < ADHD < TD, OCD
SCQ	11.34 (9.47 to 13.77)	11.90 (9.51 to 15.14)	12.98 (11.32 to 15.12)	11.6 (8.94 to 13.35)	305.01	8.18 × 10^−66^	0.61	ASD > ADHD
								OCD > TD
RBS-R	10.00 (4.00 to 20.00)	26.00 (15.00 to 40.50)	22.00 (10.75 to 36.00)	0.00 (0.00 to 2.00)	250.30	5.64 × 10^−54^	0.49	ASD, OCD > ADHD > TD
SWAN-I	6.00 (1.00 to 7.00)	5.00 (2.00 to 7.00)	1.00 (0.00 to 3.25)	0.00 (0.00 to 0.00)	224.57	2.07 × 10^−48^	0.45	ADHD > ASD > OCD > TD
SWAN-HI	3.50 (1.00 to 7.00)	3.00 (1.00 to 7.00)	0.00 (0.00 to 2.00)	0.00 (0.00 to 0.00)	174.46	1.38 × 10^−37^	0.35	ADHD, ASD > OCD > TD
TOCS	−22.00 (−48.00 to −2.00)	−5.00 (−31.50 to 6.75)	20.00 (12.00 to 33.75)	−43.50 (−59.00 to −11.50)	149.46	3.44 × 10^−32^	0.30	OCD > ASD > ADHD > TD

Abbreviations: ADHD, attention-deficit/hyperactivity disorder; ASD, autism spectrum disorder; FSIQ, full-scale intelligence quotient; NA, not applicable; OCD, obsessive-compulsive disorder; QU-TT, Queen’s University TimTrio; RBS-R, Repetitive Behaviors Scale–Revised; SCQ, Social Communication Questionnaire; SK-PF, SickKids PrismaFIT; SK-TT, SickKids TimTrio; SWAN-HI, Strengths and Weaknesses of ADHD–Symptoms and Normal Behavior Hyperactivity/Impulsivity subscale; SWAN-I, Strengths and Weaknesses of ADHD–Symptoms and Normal Behavior Inattention subscale; TD, typically developing; TOCS, Toronto Obsessive-Compulsive Scale.

^a^ Descriptions and ranges of all measures appear in the eMethods in Supplement 1.

^b^ Test statistic: Shapiro-Wilk *W* for nonnormally distributed continuous variables, 1-way analysis of variance *F* statistic for normally distributed continuous variables, χ^2^ for categorical variables, and ordinal regression χ^2^ for ordinal variables.

^c^ Effect size: eta-squared (η^2^) for continuous variables, Cramer *V* for categorical variables, and pseudo-*R*^2^ for ordinal variables.

^d^ Indication of which pairwise diagnosis differences are significant according to post hoc tests, with > and < symbols indicating the directionality of the association.

^e^ Level 1 indicates caregiver did not complete high school; level 2, high school education; level 3, associate degree; level 4, undergraduate degree; level 5, graduate or professional degree. Primary caregiver education data was available for 332 of 551 participants (74 ADHD, 131 ASD, 12 OCD, and 105 TD).

^f^ Low indicates less than $74 999; medium, $75 000 to $199 999; high, greater than $200 000. Household income was available for 286 of 551 participants (72 ADHD, 114 ASD, 8 OCD, 92 TD).

^g^ Includes Black, East Asian, Indigenous, Latino, Middle Eastern, South Asian, and Southeast Asian individuals as well as those who did not identify as 1 of the Canadian Institutes of Health Information groups (ie, other). Race and ethnicity were available for 381 of 551 participants (113 ADHD, 139 ASD, 35 OCD, 99 TD).

**Table 2.** Descriptive Statistics of the Participant Demographics and Clinical Behavioral Measures for the Healthy Brain Network Data Set, With the Corresponding Statistics Identifying Significant Differences Among the Diagnostic Groups

Measure^a^	Median (IQR)				Statistics			
	ADHD (n = 374)	ASD (n = 66)	OCD (n = 11)	TD (n = 100)	Test statistic^b^	*P* value	Effect size^c^	Significant post hoc^d^
Age, y	11.20 (9.07-13.63)	13.64 (10.83-16.04)	12.77 (11.09-14.87)	11.36 (9.36-14.22)	18.39	3.65 × 10^−4^	0.03	ASD > ADHD, TD
Sex								ASD > ADHD, OCD, TD
Male	273 (73.0)	57 (86.4)	6 (54.5)	54 (54.0)	23.7	2.95 × 10^−5^	0.21	ADHD > TD
Female	101 (27.0)	9 (13.6)	5 (45.5)	46 (46.0)				
BSMSS^e^	50.00 (40.00-59.50)	50.25 (42.00-61.00)	61.00 (48.88-62.88)	53.00 (45.75-61.00)	4.24	.24	0.01	NA
Race and ethnicity								
Minoritized race and ethnicity^f^	182 (48.7)	26 (39.4)	2 (18.2)	42 (42.0)	4.8	.19	0.10	NA
White	170 (45.5)	30 (45.5)	8 (72.7)	49 (49.0)				
Scanner								
CBIC	144 (38.5)	34 (51.5)	4 (36.4)	21 (21.0)	35.1	4.20 × 10^−6^	0.18	CBIC: ASD > ADHD > TD; SI: ADHD, ASD < TD
RU	184 (49.2)	25 (37.9)	5 (45.5)	46 (46.0)				
SI	46 (12.3)	7 (10.6)	2 (18.2)	33 (33.0)				
Head motion, mm	0.18 (0.12-0.25)	0.15 (0.11-0.26)	0.16 (0.10-0.30)	0.14 (0.11-0.22)	8.13	.04	0.01	ADHD > TD
FSIQ, mean (SD)	96.84 (15.82)	94.03 (19.65)	106.73 (19.37)	106.64 (15.47)	11.53	2.52 × 10^−7^	0.06	ASD, ADHD < TD
SCQ	7.00 (4.00-10.00)	13.00 (10.00-18.00)	4.00 (3.25-6.75)	6.00 (3.00-8.00)	92.06	7.92 × 10^−20^	0.17	ASD > ADHD, OCD, TD
								ADHD > TD
RBS-R	11.00 (2.00-38.00)	43.00 (15.00-66.00)	24.50 (16.50-51.50)	1.00 (1.00-7.00)	58.20	1.43 × 10^−12^	0.14	ASD > ADHD, OCD, TD
								ADHD > TD
SWAN-I	4.00 (1.00-7.00)	3.00 (1.00-5.75)	2.00 (0.00-3.00)	0.00 (0.00-1.00)	96.45	9.00 × 10^−21^	0.18	ADHD, ASD > TD
SWAN-HI	1.00 (0.00-3.00)	1.00 (0.00-4.00)	0.00 (0.00-0.00)	0.00 (0.00-0.00)	65.09	4.80 × 10^−14^	0.12	ADHD > OCD, TD
								ASD > TD
CBCL-OCS	3.00 (2.00-4.00)	3.00 (2.00-5.00)	3.00 (2.00-5.75)	1.00 (0.00-2.00)	91.99	8.19 × 10^−20^	0.17	ADHD, ASD, OCD > TD

Abbreviations: ADHD, attention-deficit/hyperactivity disorder; ASD, autism spectrum disorder; BSMSS, Barratt Simplified Measure of Social Status; CBCL-OCS, Child Behavior Checklist obsessive-compulsive subscale; CBIC, CitiGroup Cornell Brain Imaging Center; FSIQ, full-scale intelligence quotient; NA, not applicable; OCD, obsessive-compulsive disorder; RBS-R, Repetitive Behaviors Scale–Revised; RU, Rutgers University; SCQ, Social Communication Questionnaire; SI, Staten Island; SWAN-HI, Strengths and Weaknesses of ADHD–Symptoms and Normal Behavior Hyperactivity/Impulsivity subscale; SWAN-I, Strengths and Weaknesses of ADHD–Symptoms and Normal Behavior Inattention subscale; TD, typically developing.

^a^ Descriptions and ranges of all measures appear in the eMethods in Supplement 1.

^b^ Test statistic: Shapiro-Wilk *W* for nonnormally distributed continuous variables, 1-way analysis of variance *F* statistic for normally distributed continuous variables, χ^2^ for categorical variables, and ordinal regression χ^2^ for ordinal variables.

^c^ Effect size: eta-squared (η^2^) for continuous variables, Cramer *V* for categorical variables, and pseudo-*R*^2^ for ordinal variables.

^d^ Indication of which pairwise diagnosis differences are significant according to post hoc tests, with > and < symbols indicating the directionality of the association.

^e^ The range for BSMSS is 8 to 66, with higher scores indicating a higher social status.

^f^ Includes American Indian or Alaskan Native, Asian, Black, Hispanic, Native Hawaiian or other Pacific Islander, and those who selected 2 or more races/ethnicities and those who did not identify as one of the US Census guideline categories. Data were available for 509 of 511 participants (352 ADHD, 56 ASD, 10 OCD, 91 TD).

Compared with the POND sample, the HBN sample had a significantly higher proportion of ADHD and a lower proportion of ASD and OCD diagnoses (χ^2^ = 197.74; *P* < .001; *V* = 0.42). Furthermore, the HBN sample had significantly more participants belonging to minoritized racial and ethnic groups (χ^2^ = 6.50; *P* = .01; *V* = 0.09), lower full-scale IQ (*U* = 1.15 × 10^5^; *P* = .01; η^2^ = 7.44 × 10^−3^), and fewer social communication difficulties (*U* = 1.26 × 10^5^; *P* = .03; η^2^ = 1.71 × 10^−3^) and hyperactivity and impulsivity problems, measured by the Strengths and Weaknesses ADHD Symptoms and Normal Behavior Hyperactivity/Impulsivity subscale (SWAN-H/I; *U* = 1.09 × 10^5^; *P* < .001; η^2^ = 0.03). eTable 4 in Supplement 1 includes full details.

### Clustering

**Figure 2.**
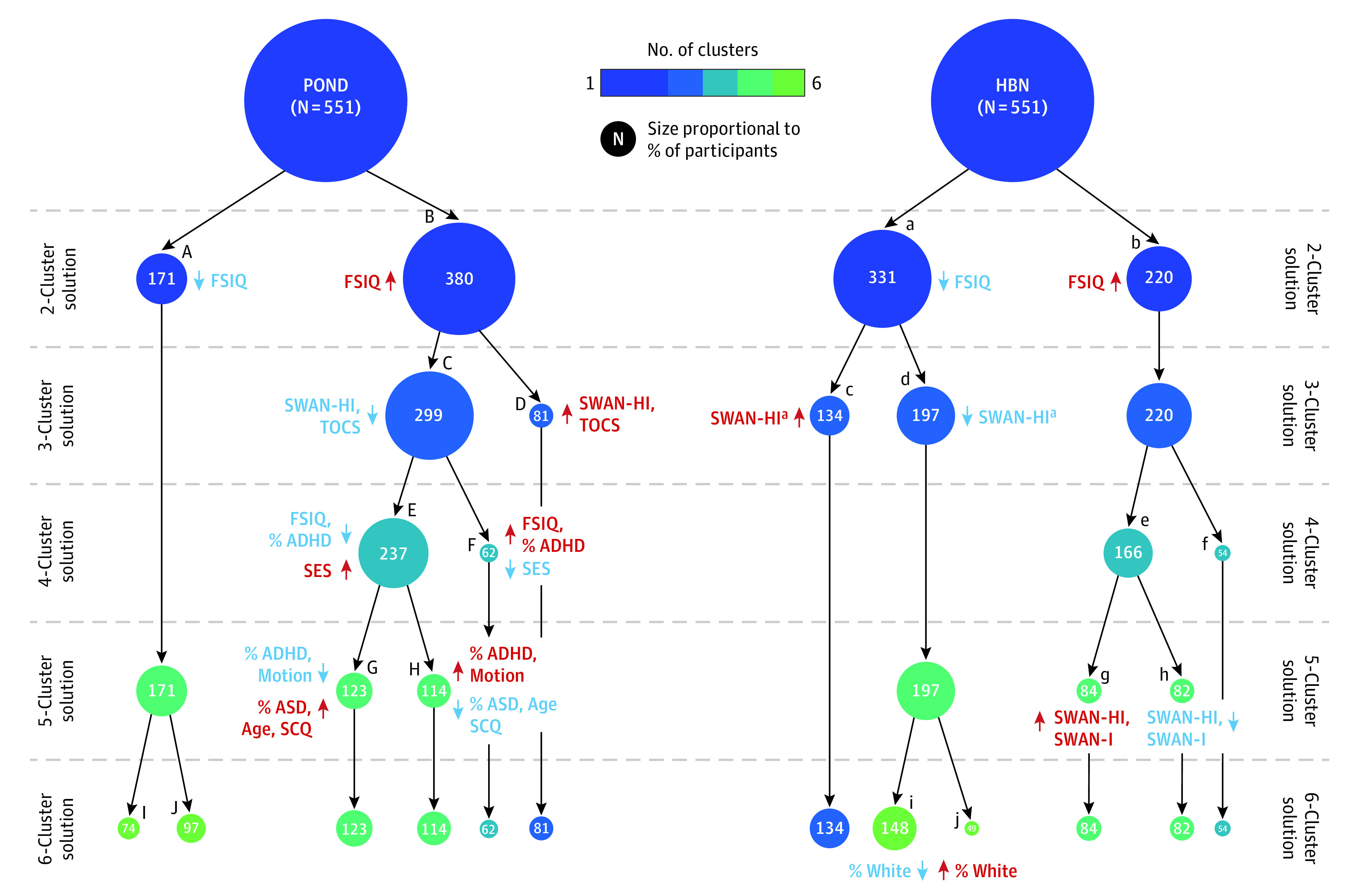
Clustering Results Dendrograms showing the emergence of the 6 clusters in the Province of Ontario Neurodevelopmental (POND) network and Healthy Brain Network (HBN) data sets. The circle colors correspond to the number of specified clusters, and their size is proportional to the percentage of participants included in the subgroup. For each layer of the dendrogram, Mann-Whitney *U* or *t *tests were used to identify pairwise differences in sample characteristics between the leaf clusters (letters A through J in uppercase and lowercase for POND and HBN, respectively); the directionality of significant (*P* < .05) effect sizes are identified, with red indicating the leaf cluster with an increase in the clinical measure and blue indicating a decrease. ADHD indicates attention-deficit/hyperactivity disorder; ASD, autism spectrum disorder; FSIQ, full-scale intelligence quotient; SCQ, Social Communication Questionnaire; SES, socioeconomic status; SWAN-HI, Strengths and Weaknesses of ADHD—Symptoms and Normal Behavior Hyperactivity/Impulsivity subscale; SWAN-I, Strengths and Weaknesses of ADHD—Symptoms and Normal Behavior Inattention subscale; TOCS, Toronto Obsessive-Compulsive Scale. ^a^Corrected *P* = .06.

The Calinski-Harabasz index indicated that the 6- and 10-cluster solutions were optimal for POND and HBN, respectively (eFigure 2 in Supplement 1). Visual representations of the emergence of the 6 clusters in the POND and HBN data sets are presented in Figure 2, and statistical results appear in eTables 5 to 8 in Supplement 1. Significant differences between the leaf clusters in network-averaged measures of segregation and integration for each layer in the POND and HBN dendrograms are shown in eFigure 3 and eTables 9 to 12 in Supplement 1.

**Figure 3.**
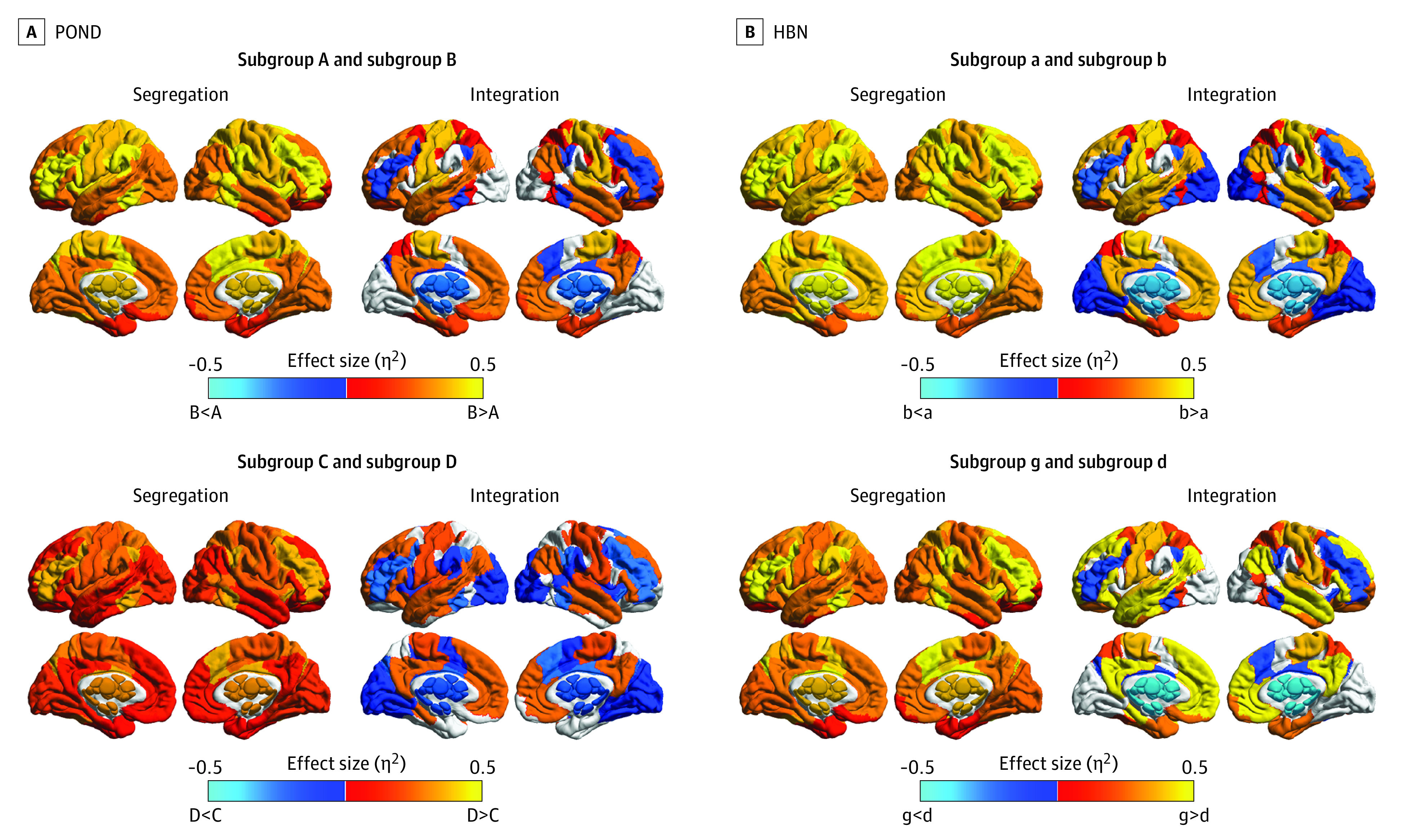
Between-Subgroup Differences in Brain Measures The effect size of significant (ie, corrected *P* < .05) differences in network-averaged measures of segregation and integration between the Province of Ontario Neurodevelopmental (POND) and Health Brain Network (HBN) subgroups that differed in intelligence and hyperactivity and impulsivity.

For both POND and HBN data sets, the 2-cluster solution split the sample into 2 groups (POND: subgroups A and B; HBN: subgroups a and b). Subgroups B and b had increased segregation in all resting-state networks; increased integration in the somatomotor, dorsal attention, limbic and default mode networks; and decreased integration in the frontoparietal control network and subcortical regions compared with subgroups A and a (Figure 3A and B). Although integration of the visual network was also observed to be decreased in the HBN data set, this was not replicated in the POND data set. A difference in IQ scores was observed (full-spectrum IQ, POND: *U* = 2.86 × 10^4^; *P* = .04; η^2^ = 0.01; full-spectrum IQ, HBN: *t* = −2.37; *P* = .02; η^2^ = 0.01), with subgroups B and b having increased IQ scores compared with subgroups A and a (median [IQR] IQ scores, B vs A: 104.00 [92.00-114.00] vs 100.00 [88.00-110.00]; mean [SD] IQ scores, b vs a: 100.63 [16.98] vs 97.08 [16.55]) (eFigure 4 in Supplement 1). In both data sets, there were no differences in the proportion of each diagnosis between the leaves.

In subsequent layers of the dendrogram, consistent results were also observed with respect to cluster splits resulting in differences in hyperactivity and impulsivity symptoms as measured by SWAN-HI. In the third layer of the POND dendrogram, a significant difference in SWAN-HI (*U* = 1.19 × 10^4^; *P* = .01; η^2^ = 0.02) was identified between the 2 leaf subgroups (C and D), with subgroup D having increased hyperactivity and impulsivity traits compared with subgroup C (median [IQR], 2.50 [0.00-7.00] vs 1.00 [0.00-5.00]). In the HBN dendrogram, the leaves in the third layer (subgroups c and d) also showed a difference in hyperactivity and impulsivity, but the difference was not statistically significant (*U* = 1.13 × 10^4^; *P* = .06; η^2^ = 0.01). A significant difference in symptoms was observed in the fifth layer (subgroups g and h: *U* = 2.68 × 10^3^; *P* = .02; η^2^ = 0.03). To contrast with POND, the 4 HBN subgroups (c, d, g, and h) were compared, and a difference in hyperactivity and impulsivity problems was identified (*W* = 10.81; *P* = .01; η^2^ = 0.02), with post hoc tests identifying that subgroup g had significantly higher hyperactivity and impulsivity compared with subgroup d (median [IQR], 1.00 [0.00-4.00] vs 0.00 [0.00-2.00]; corrected *P* = .02). Thus, POND subgroup D and HBN subgroup g were identified as having increased SWAN-HI scores compared with POND subgroup C and HBN subgroup d (eFigure 4 in Supplement 1). Differences in the brain measures were compared between the 2 subgroups for both POND and HBN (Figure 3C and D; eTable 13 in Supplement 1). In both data sets, we observed increased segregation in all networks in the subgroup with higher hyperactivity and impulsivity, with the largest effect sizes occurring in the somatomotor and default mode networks in both data sets, along with the dorsal attention network in HBN. Increased integration in the motor and default mode networks and decreased integration in the frontoparietal and subcortical networks were also observed in both data sets. Despite these subgroups differing in symptoms associated with ADHD, we observed no differences in the proportion of each diagnosis between the subgroups in either data set.

In subsequent layers of the POND dendrogram, we observed differences in the proportion of diagnoses between the leaf clusters in the 4- and 5-cluster solutions. However, these diagnostic differences were not replicated in the HBN data set.

## Discussion

In this study, we used measures derived from the brain’s functional networks to identify data-driven transdiagnostic subgroups of children and adolescents with and without neurodevelopmental conditions to characterize the heterogeneity across the conditions; these data-driven subgroups were then described using demographic and clinical indices. We identified subgroups in 2 independently collected data sets—POND and HBN—and, focusing on findings that were present in both cohorts, found subgroups that differed in intelligence and hyperactivity and impulsivity symptoms but not diagnosis.

Research on neurodevelopmental conditions has classically operated under the assumption that diagnostic labels are the ground truth. However, there is increasing awareness of the biological and symptom heterogeneity within conditions and overlap across conditions, which has raised concerns about the appropriateness of service provision systems in health care that are based on diagnostic labels.^50,51^ Our work joins the growing body of literature supporting transdiagnostic approaches for accommodating the variability and complexity of these conditions and provides support for categorizing individuals on biology to identify better targets for treatments and interventions.

Furthermore, to our knowledge, we are the first to replicate our discovered transdiagnostic subgroups across 2 independently collected data sets, showing that similar subgroups with specific brain signatures can be identified that are accompanied by replicable phenotypic differences. With a mounting body of work challenging the reproducibility of brain-behavior relations^13,34^ and brain-based subtyping,^52^ replicating clustering results is essential to establish the robustness necessary to translate the groupings to clinical settings, particularly given the heterogeneity in both brain and behavior in neurodevelopmental conditions. While previous studies have established transdiagnostic subgroupings using neuroimaging,^9,12,27,28,30,31,32,53^ these results should be interpreted cautiously in the absence of replication. We explicitly address their shortcomings by evaluating subgroup replicability in 2 independent data sets.

Our discovered brain-based subgroups spanned the spectrum of neurodiversity, including typical development, and do not align with existing categorical boundaries. Specifically, we identified subgroups with similar biology that differed significantly in intelligence and hyperactivity and impulsivity problems yet showed no consistent alignment with the current diagnostic categories. The identification of neurobiologically defined subgroups that align poorly with the current behavior-based diagnostic categories contributes to the work criticizing the lack of correspondence of these categorical descriptors with biology.^9,11,27,28,30,32,53^ The TD individuals were also spread across all identified brain-based subgroups, emphasizing that an overlap in neurobiology exists not only across conditions, but also across typical development, aligning with emerging studies highlighting the similarities, rather than differences, in resting-state brain function between populations with neurodevelopmental conditions and TD populations.^9,10,54^

We identified replicable subgroups differing in intelligence, between which no differences in diagnosis (including those with no diagnosis) were observed. The distribution of each diagnosis across the subgroups aligned with the diversity in traits observed in neurodevelopmental conditions. For example, although neurodevelopmental conditions demonstrate overall reduced intelligence compared with their TD peers,^55,56,57^ those with ASD have been shown to also have a higher probability of scoring in the superior intelligence range.^55^ We also identified subgroups in both data sets who differed in hyperactivity and impulsivity traits. Even though these traits are traditionally considered characteristic of ADHD, we did not observe a difference in proportion of diagnostic categorization between these subgroups in either data set. This is consistent with the finding that hyperactivity and impulsivity are shared characteristics across neurodevelopmental conditions.^58,59,60^ The identification of subgroups that differ in these behavioral characteristics, rather than by diagnosis, supports using continuous measures of behavior to study neurodevelopmental differences rather than relying on the current discrete categorical categories.

These differences in intelligence and hyperactivity and impulsivity symptoms were accompanied by pervasive differences in the brain’s functional segregation and integration. Networks involved in intelligence are distributed throughout the brain to support distinct information processing stages.^61^ Similarly, there was no convergence in the spatial patterns of functional brain connectivity associated with ADHD in the literature,^17^ and ADHD traits have been reported to be more associated with brainwide connectivity than with local connectivity.^62^ Thus, our findings support the distributed involvement of brain regions in intelligence and hyperactivity and impulsivity symptoms.

We observed increased segregation in all brain networks coupled with predominantly increased integration in the subgroups with increased intelligence. Simultaneous increases in both segregation and integration occurs throughout development as hubs in the brain’s network shift from primary to cognitive brain regions to support cognitive development.^63,64^ Opposite to the other networks, the integration of the frontoparietal control and subcortical networks was decreased in the subgroup with increased intelligence. The specific pattern of involvement in these brain networks in intelligence aligns with the parieto-frontal integration theory of intelligence^61^ that has been extended to include subcortical structures.^65,66^

The subgroup with increased hyperactivity and impulsivity demonstrated widespread increased segregation and patterns of both increased (eg, motor) and decreased (eg, subcortical) integration. To our knowledge, no study has identified alterations in the topography of the brain’s resting-state functional network that are specific to hyperactivity and impulsivity in ADHD. The few studies examining the associations between hyperactivity and impulsivity and functional connectivity have implicated connections between striatal regions and regions in the motor network.^67,68,69^ The significant and opposite associations of integration we observed in these networks reinforces their specific involvement to hyperactivity and impulsivity symptoms.

### Limitations

This study has limitations. Our study focused on brain function; however, differences between individuals with and without neurodevelopmental conditions have been observed in measures of both brain function and structure as well as in other domains. Thus, our study is limited by focusing on only one aspect of the brain, and our identified subgroups may not be homogeneous in other measures. There was a limited number of participants with OCD compared with the other diagnostic groups, and most behavioral measures used to characterize the data-driven subgroups were parent-rated reports, which may not be impartial. Furthermore, our age range is restricted to children and adolescents, a developmental period when significant changes are occurring; future work should extend the age range into adulthood and examine how the identified subgroups change throughout development. We have also only used a single 5-minute resting-state scan from the HBN data set when 2 were available and passed quality control; future work could evaluate the within-participant stability of our subgroups between multiple resting-state scans. The removal of nuisance covariates (age, sex, head motion, and acquisition scanner) also may inadvertently remove signal of interest yet was necessary to ensure subgroups were not defined by these covariates. Additionally, the study design was cross-sectional, and future studies should incorporate longitudinal data to examine the stability of the clusters over time. It is important to note that the findings of this study are based on neurobiological profiles quantified through measurements of brain function. As such, these results do not reflect broader considerations for existing diagnostic categories including issues of self-identity and service provision. Consultations and partnerships with neurodiverse populations are needed to appropriately contextualize and translate these findings into clinical practice. We recognize the different language preferences for referring to autistic identity (identity-first language and person-first language). We use both in this paper to reflect the diversity of perspectives.

## Conclusions

To our knowledge, this is the first study to identify transdiagnostic subgroups replicated across 2 independent data sets. With the reliability of associations between brain and behavior being increasingly questioned in the literature, stratification techniques are a useful way of increasing power by identifying more homogeneous subgroups within the sample to target treatments and interventions. The replication of exact subgroups across different samples with varying diagnostic and behavioral characteristics is an essential step in ensuring robustness prior to implementing the groupings into clinical settings. Finally, our study suggests that homogeneity in neurobiology transcends diagnostic boundaries, promoting a shift in the research community away from classic case-control designs that rely on diagnostic categories, which have increasingly been shown not to reflect distinct biological and phenotypic constructs.

## Notes

Supplement 1.
eMethods.
**eFigure 1.** Cortical and Subcortical Parcellation Used in the Analysis **eFigure 2.** The Calinski-Harabasz Index for Each Clustering Solution for the POND (A) and HBN (B) Clustering **eFigure 3.** POND (A) and HBN (B) Dendrograms **eFigure 4.** Distributions of Intelligence and Hyperactivity/Impulsivity, Separated for Males and Females, for the Subgroups Showing Replicable Differences **eTable 1.** MRI Protocols for the T1-Weighted and Resting-State Data Acquired Using the 3 Scanners **eTable 2.** Normality Test Statistics for the Continuous Measures Describing the POND and HBN Sample Characteristics and the Clinical Behavioural Measures **eTable 3.** Race and Ethnicity Data for the POND and HBN Data Sets **eTable 4.** Descriptive Statistics of the Participant Demographics and Clinical Behavioural Measures Comparing the POND and HBN Data Sets, With Corresponding Statistics Identifying Significant (p<0.05) Differences Between the Data Sets, With the Directionality of the Difference Highlighted **eTable 5.** Descriptive Statistics of the Participant Demographics and Clinical Behavioural Measures for Each Leaf Cluster for Each Layer of the POND Dendrogram **eTable 6.** Descriptive Statistics of the Participant Demographics and Clinical Behavioural Measures for Each Leaf Cluster for Each Layer of the HBN Dendrogram **eTable 7.** Statistical Details of the Mann Whitney U and t Tests and χ^2^ Tests Examining Differences in Sample Characteristics Between the Leaf Clusters in Each Layer of the POND Dendrogram **eTable 8.** Statistical Details of the Mann Whitney U and t Tests and χ^2^ Tests Examining Differences in Sample Characteristics Between the Leaf Clusters in Each Layer of the HBN Dendrogram **eTable 9.** Descriptive Statistics of the Network-Averaged Measures of Segregation and Integration for Each Leaf Cluster for Each Layer of the POND Dendrogram **eTable 10.** Descriptive Statistics of the Network-Averaged Measures of Segregation and Integration for Each Leaf Cluster for Each Layer of the HBN Dendrogram **eTable 11.** Statistical Details of the Tests Examining Differences in the Network-Averaged Measures of Segregation and Integration Between the Leaf Clusters in Each Layer of the POND Dendrogram **eTable 12.** Statistical Details of the Tests Examining Differences in the Network-Averaged Measures of Segregation and Integration Between the Leaf Clusters in Each Layer of the HBN Dendrogram **eTable 13.** Statistical Details of the Tests Examining Differences in the Network-Averaged Measures of Segregation and Integration Between the Leaf Clusters From the HBN Dendrogram That Differed in Hyperactivity/Impulsivity Problems (Subgroup d and g)
eReferences.

Supplement 2.
Data Sharing Statement
