# Supplementary material for: Identifying Replicable Subgroups in Neurodevelopmental Conditions Using Resting-State Functional Magnetic Resonance Imaging Data
Source: JAMA Netw Open. 2023 Mar 13;6(3):e232066. doi: 10.1001/jamanetworkopen.2023.2066 (PMC10011941; doi:10.1001/jamanetworkopen.2023.2066)
Supplement: Supplement 1. — eMethods. eFigure 1. Cortical and Subcortical Parcellation Used in the Analysis eFigure 2. The Calinski-Harabasz Index for Each Clustering Solution for the POND (A) and HBN (B) Clustering eFigure 3. POND (A) and HBN (B) Dendrograms eFigure 4. Distributions of Intelligence and Hyperactivity/Impulsivity, Separated for Males and Females, for the Subgroups Showing Replicable Differences eTable 1. MRI Protocols for the T1-Weighted and Resting-State Data Acquired Using the 3 Scanners eTable 2. Normality Test Statistics for the Continuous Measures Describing the POND and HBN Sample Characteristics and the Clinical Behavioural Measures eTable 3. Race and Ethnicity Data for the POND and HBN Data Sets eTable 4. Descriptive Statistics of the Participant Demographics and Clinical Behavioural Measures Comparing the POND and HBN Data Sets, With Corresponding Statistics Identifying Significant (p<0.05) Differences Between the Data Sets, With the Directionality of the Difference Highlighted eTable 5. Descriptive Statistics of the Participant Demographics and Clinical Behavioural Measures for Each Leaf Cluster for Each Layer of the POND Dendrogram eTable 6. Descriptive Statistics of the Participant Demographics and Clinical Behavioural Measures for Each Leaf Cluster for Each Layer of the HBN Dendrogram eTable 7. Statistical Details of the Mann Whitney U and t Tests and χ2 Tests Examining Differences in Sample Characteristics Between the Leaf Clusters in Each Layer of the POND Dendrogram eTable 8. Statistical Details of the Mann Whitney U and t Tests and χ2 Tests Examining Differences in Sample Characteristics Between the Leaf Clusters in Each Layer of the HBN Dendrogram eTable 9. Descriptive Statistics of the Network-Averaged Measures of Segregation and Integration for Each Leaf Cluster for Each Layer of the POND Dendrogram eTable 10. Descriptive Statistics of the Network-Averaged Measures of Segregation and Integration for Each Leaf Cluster for Each Layer of the HBN Dendrogram eTab [file jamanetwopen-e232066-s001.pdf]

Supplemental Online Content

Vandewouw MM, Brian J, Crosbie J, et al. Identifying replicable subgroups in neurodevelopmental conditions using resting-state functional magnetic resonance imaging data. *JAMA Netw Open.* 2023;6(3):e232066.
doi:10.1001/jamanetworkopen.2023.2066

eMethods.

eFigure 1. Cortical and Subcortical Parcellation Used in the Analysis

eFigure 2. The Calinski-Harabasz Index for Each Clustering Solution for the POND (A) and HBN (B) Clustering

eFigure 3. POND (A) and HBN (B) Dendrograms

eFigure 4. Distributions of Intelligence and Hyperactivity/Impulsivity, Separated for Males and Females, for the Subgroups Showing Replicable Differences

eTable 1. MRI Protocols for the T1-Weighted and Resting-State Data Acquired Using the 3 Scanners

eTable 2. Normality Test Statistics for the Continuous Measures Describing the POND and HBN Sample Characteristics and the Clinical Behavioural Measures

eTable 3. Race and Ethnicity Data for the POND and HBN Data Sets

eTable 4. Descriptive Statistics of the Participant Demographics and Clinical Behavioural Measures Comparing the POND and HBN Data Sets, With Corresponding Statistics Identifying Significant ($p<0.05$) Differences Between the Data Sets, With the Directionality of the Difference Highlighted

eTable 5. Descriptive Statistics of the Participant Demographics and Clinical Behavioural Measures for Each Leaf Cluster for Each Layer of the POND Dendrogram

eTable 6. Descriptive Statistics of the Participant Demographics and Clinical Behavioural Measures for Each Leaf Cluster for Each Layer of the HBN Dendrogram

eTable 7. Statistical Details of the Mann Whitney U and t Tests and χ^2 Tests Examining Differences in Sample Characteristics Between the Leaf Clusters in Each Layer of the POND Dendrogram

eTable 8. Statistical Details of the Mann Whitney U and t Tests and χ^2 Tests Examining Differences in Sample Characteristics Between the Leaf Clusters in Each Layer of the HBN Dendrogram

eTable 9. Descriptive Statistics of the Network-Averaged Measures of Segregation and Integration for Each Leaf Cluster for Each Layer of the POND Dendrogram

© 2023 Vandewouw MM et al. *JAMA Network Open.*

eTable 10. Descriptive Statistics of the Network-Averaged Measures of Segregation and Integration for Each Leaf Cluster for Each Layer of the HBN Dendrogram

eTable 11. Statistical Details of the Tests Examining Differences in the Network-Averaged Measures of Segregation and Integration Between the Leaf Clusters in Each Layer of the POND Dendrogram

eTable 12. Statistical Details of the Tests Examining Differences in the Network-Averaged Measures of Segregation and Integration Between the Leaf Clusters in Each Layer of the HBN Dendrogram

eTable 13. Statistical Details of the Tests Examining Differences in the Network-Averaged Measures of Segregation and Integration Between the Leaf Clusters From the HBN Dendrogram That Differed in Hyperactivity/Impulsivity Problems (Subgroup d and g)

eReferences.

This supplemental material has been provided by the authors to give readers additional information about their work.

© 2023 Vandewouw MM et al. *JAMA Network Open.*

eMethods.

Participants

The POND dataset consists of children and adolescents who are either typically developing (absence of neurodevelopmental, neurological, or neurodevelopmental diagnoses, prematurity, and first-degree family member with a neurodevelopmental condition), or who have a diagnosis of ASD, ADHD, or OCD (confirmed with expert clinical judgement and diagnostic tests[1–4]). Data for the current study was collected at two of the participating institutions: the Hospital for Sick Children (Toronto, Ontario, Canada) and Queen's University (Kingston, Ontario, Canada). The POND study was approved by each participating institution's research ethics boards; written informed consent or assent was obtained from the primary caregiver or participant where appropriate.

For the HBN dataset, data were collected at three of the participating institutions in the New York City area (the CitiGroup Cornell Brain Imaging Center (CBIC), Rutgers University (RU), and a mobile site in Staten Island (SI)). The Child Mind Institute's research ethics board approved the study; informed consent and verbal assent was obtained from the primary caregiver and participant, respectively. The consensus clinical diagnosis using the DSM-5[5] was used to identify individuals with ASD, ADHD, or OCD; those who did not receive a neurodevelopmental diagnosis, nor any other diagnosis, were considered TD.

Race and ethnicity were reported to evaluate the racial and ethnic distribution in both the POND and HBN cohorts, and to identify whether the identified subgroups differed in their distributions. For the POND dataset, self/parent-reported race and ethnicity data was collected on 386 of the participants; categories (Black, East Asian, Indigenous, Latino, Middle Eastern, Other, South Asian, Southeast Asian, and White) were determined according to the Canadian Institute for Health Information (CIHI) standards. Participants were classified into multiple categories if they were of mixed race; those who did not identify as one of the CIHI groups were categorized as "Other". For the HBN dataset, self/parent-reported race and ethnicity data was available for 509 participants, and categories were defined according to the United States census

© 2023 Vandewouw MM et al. *JAMA Network Open.*

guidelines (American Indian/Alaskan Native, Asian, Black, Hispanic, Indian, Two or More (Mixed), Native Hawaiian/Other Pacific Islander, Native American Indian, Other, and White). Participants of mixed race were classified as such, and thus participants were only assigned to one category; those who did not identify as one of the census groups were categorized as “Other”. Due to low sample size, categories for both datasets were collapsed into minoritized racial and ethnic group and white for statistical tests, with full details provided in **Supplemental Table 3**; categories with no individuals were excluded from the table.

Socio-economic status (SES) for the POND dataset was determined using the highest level of education achieved by the primary caregiver (323 participants; Level 1: did not complete high school, Level 2: high school education, Level 3: associate degree, Level 4: undergraduate degree, Level 5: graduate/professional degree) and household income (287 participants; Low: <$74,999, Medium: $75,000 – $199,999, High: <$200,000). For the HBN dataset, Barratt Simplified Measure of Social Status (BSMSS) was obtained on 540 participants.

Clinical behavioural measures

For the POND and HBN datasets, full-scale IQ (FSIQ) was assessed with Wechsler[6–10] or Stanford-Binet[11] scales (range: 40-160, with higher scores indicating higher intelligence). ASD-like traits were measured using Social Communication Questionnaire[12] (range: 0-39, with higher scores indicating more severe difficulties with social communication), and repetitive behaviours were measured using the Repetitive Behaviours Scale – Revised[13] (range: 0-129, with higher scores indicating more severe difficulties with repetitive behaviours). The Strengths and Weaknesses of ADHD-symptoms and Normal Behaviour[14] rating scale inattention subscale (SWAN-I) was used to measure inattention (range: 0-9, with higher scores indicating more severe difficulties with inattention), while hyperactivity and impulsivity was measured using the SWAN hyperactivity/impulsivity subscale (SWAN-HI) (range: 0-9, with higher scores indicating more severe difficulties with hyperactivity/impulsivity). For the POND dataset, obsessive-compulsive traits were measured using the Toronto Obsessive-Compulsive Scale (TOCS) (range: -63 to 63, with higher scores indicating more severe difficulties

© 2023 Vandewouw MM et al. *JAMA Network Open.*

with obsessive-compulsive traits). A direct measure of obsessive-compulsive traits was not obtained on the HBN participants, and thus the obsessive-compulsive subscale of the CBCL (CBCL-OCS[15]) was used (range: 0-16, with higher scores indicating more severe difficulties with obsessive-compulsive traits), which shows moderate correlation with TOCS[16].

Data acquisition

Neuroimaging data from POND were acquired on one of three Siemens 3T MRI scanners; for each participant, a T1-weighted image and five minutes of resting-state data were obtained while the participant viewed a movie of their choosing or *Inscapes*, a naturalistic movie paradigm[17]. Neuroimaging data from HBN were acquired at one of three sites; for each participant, a T1-weighted image and resting-state data were obtained while the participant was instructed to focus on a fixation cross. Ten minutes of resting-state data were acquired, broken up into two five-minute runs. To best conform with the five-minute POND dataset, for HBN participants with two five-minute runs, a single run was selected by using propensity score matching to minimize the difference in head motion between the two datasets (see section ***Propensity score matching*** in the eMethods). The MRI protocols for each scanner are summarized in **eTable 1**.

Preprocessing

The neuroimaging data were preprocessed using *fMRIPrep*[18,19], a preprocessing tool based in Nipype[20,21] that consists of an anatomical and functional pipeline. For the anatomical pipeline, each participant's T1-weighted image was corrected for intensity non-uniformity[22] and skull-stripped using the OASIS template with Advance Normalization Tools (ANTs) to generate a brain mask. FreeSurfer[23] was used to reconstruct brain surfaces, which were used to refine the brain mask using a custom variation of Mindboggle[24]. ANTs nonlinear registration[25] was used spatially normalize the brain-extracted image to a pediatric template[26,27]. FMRIB's Software Library (FSL) was used to segment the brain-extracted normalized image into cerebrospinal fluid (CSF), white matter (WM), and gray matter[28].

For the functional pipeline, each participant's data was slice-time and motion corrected using Analysis of Functional NeuroImages (AFNI[29]) and FSL[30] software,

© 2023 Vandewouw MM et al. *JAMA Network Open.*

respectively. Fieldmap-less distortion correction was performed by co-registering the data to the corresponding T1-weighted image with intensity inverted[31,32], constrained with an average fieldmap template[33], implemented with ANTs. The resulting data were co-registered to the T1-weighted image using boundary-based registration with six degrees of freedom implemented in FreeSurfer[34]. The motion-correcting, functional-to-anatomical, and anatomical-to-template transformations were concatenated and applied in a single step with ANTs using Lanczos interpolation. Framewise displacement (FD[35]) and the standardized derivative of root mean square variance over voxels (DVARS[35]) was computed using the implementation in Nipype; mean FD across all frames was used as a measure of head motion in the analyses. Participants with more than 1/3 of their frames exceeding the recommended threshold (FD: 0.5mm, DVARS: 1.5) were excluded from all subsequent analyses. Following *fMRIPrep*, the functional data was cleaned of nuisance signals. The six motion parameters from motion correction and signal contributions from the white matter and CSF, along with their derivative and quadratic terms, were regressed from the data while simultaneously performing high-pass temporal filtering (0.008Hz) using AFNI[29,36].

Propensity score matching

Propensity score matching was used to ensure the two datasets did not differ in age, sex, and motion. Propensity scores were computed as the predicted response of a multiple logistic regression model with age, sex, and motion as independent variables and dataset as the dependent variable. A modified version of nearest-neighbour, one-to-one matching was performed to select a single five-minute run of the HBN data when two were available. For each POND participant that passed quality control (*N*=592), HBN datasets with propensity scores within a pre-specified tolerance (set to a quarter of the standard deviation of the scores[37]) were identified as eligible matches. Nearest-neighbour HBN matches were drawn for each POND participant in a randomized order; once on run of an HBN participant was selected as a match, the remaining run was removed from the list of eligible matches. This procedure was repeated (*N*=100), each time with a new randomized order, and the solution which maximized the final sample size was selected.

© 2023 Vandewouw MM et al. *JAMA Network Open.*

Nuisance covariates

To account for the influence of scanner, age, and sex, a two-step approach was employed. The first step corrected the connectome data for the different acquisition scanner (see **eTable 1**) using ComBat harmonization[38]. During ComBat, no biological variables were included as fixed effects to preserve, and the default parametric prior method was used in the empirical Bayes procedure. Next, the age and motion variables (linear, quadratic, and cubic to account for nonlinear developmental trajectories[39,40]) along with sex were regressed from each connection. Due to the non-normality of motion, the data were log-transformed prior to the regression. Both steps were applied on the pooled POND and HBN datasets to ensure no biases were introduced if applied separately, and repeated subsampling (randomly selecting 63.2% of the sample for each iteration, performed over 10,000 iterations) was employed to increase robustness[41,42].

Clustering

Similarity Network Fusion (SNF) depends on two free hyperparameters which can produce different clustering solutions: μ, the scaling parameter in the weighted similarity kernel, and K, the number of nearest neighbours used in both similarity matrix construction and fusion. Hyperparameters were optimized using the methods described in Markello et al.[44], exploring the hyperparameter space constructed using 100 logarithmically spaced values of both μ and K between their recommended ranges ($\mu \in [0.3, 0.8]$, $K \in [10,30]$, 10,000 unique combinations). A participant co-assignment matrix was then generated by computing the percentage of times two participants were clustered in the same subgroup across cluster solutions that were stable across the hyperparameter exploration.

eFigure 1. Cortical and Subcortical Parcellation Used in the Analysis

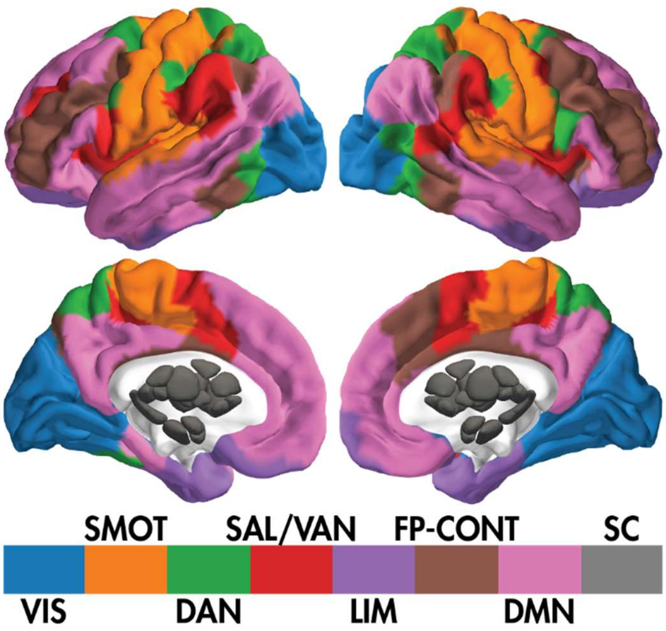

© 2023 Vandewouw MM et al. *JAMA Network Open.*

eFigure 2. The Calinski-Harabasz Index for Each Clustering Solution for the POND (A) and HBN (B) Clustering

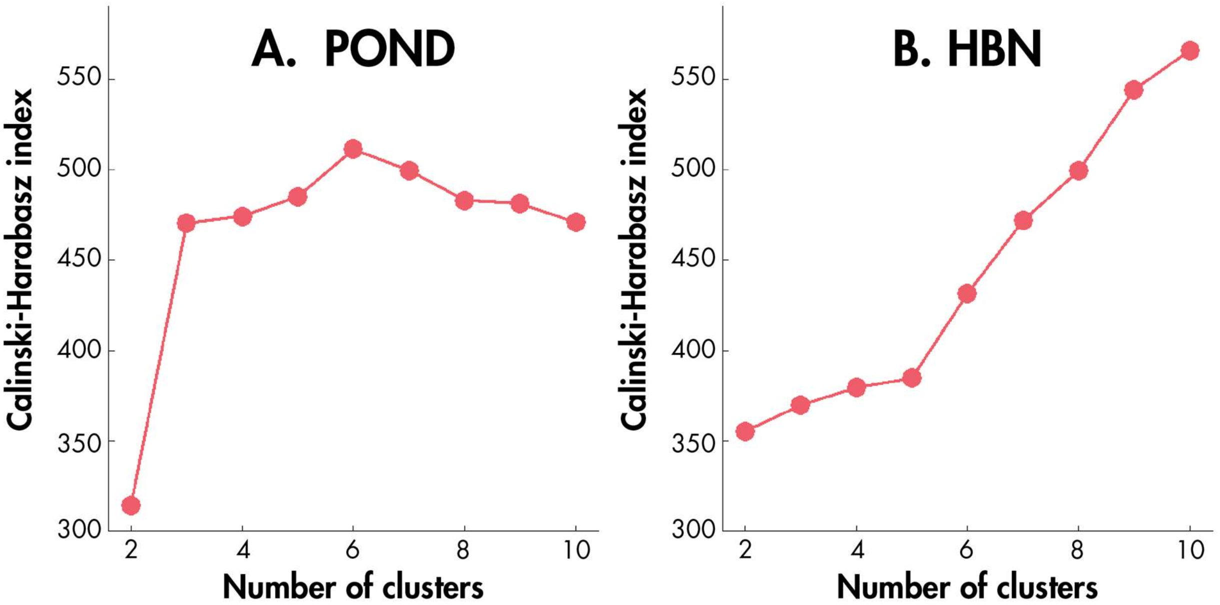

POND: Province of Ontario Neurodevelopmental Network; HBN: Healthy Brain Network.

© 2023 Vandewouw MM et al. *JAMA Network Open.*

eFigure 3. POND (A) and HBN (B) Endrograms

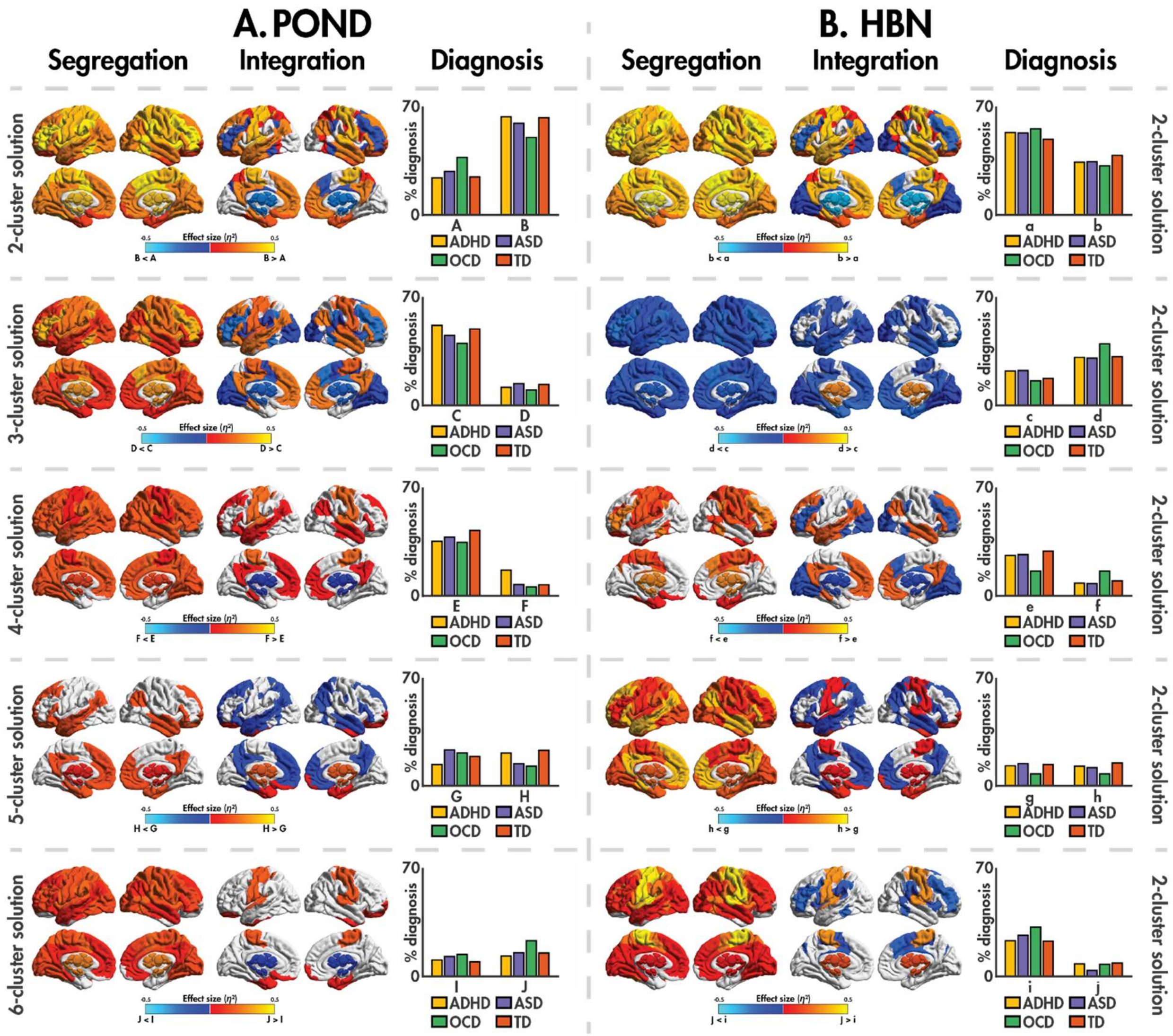

For each layer of the POND (A) and HBN (B) dendrograms, Mann-U Whitney or t-tests were used to identify pairwise differences in network-averaged measures of segregation and integration between the leaf clusters. The effect size of significant (pcorr<0.05) differences between pairs of leaf clusters are presented, showing which networks were driving the split of the root cluster. The percentage distribution of each diagnosis (yellow: ADHD, purple: ASD, green: OCD, red: TD) is shown for each of the leaf clusters. POND: Province of Ontario Neurodevelopmental Network; HBN: Healthy Brain Network; ADHD: attention-deficit/hyperactivity disorder; ASD: autism spectrum disorder; OCD: obsessive-compulsive disorder; TD: typically developing.

© 2023 Vandewouw MM et al. *JAMA Network Open.*

eFigure 4. Distributions of Intelligence (POND: A, HBN: B) and Hyperactivity/Impulsivity (POND: C, HBN: D), Separated for Males (M; blue) and Females (F; pink), for the Subgroups Showing Replicable Differences

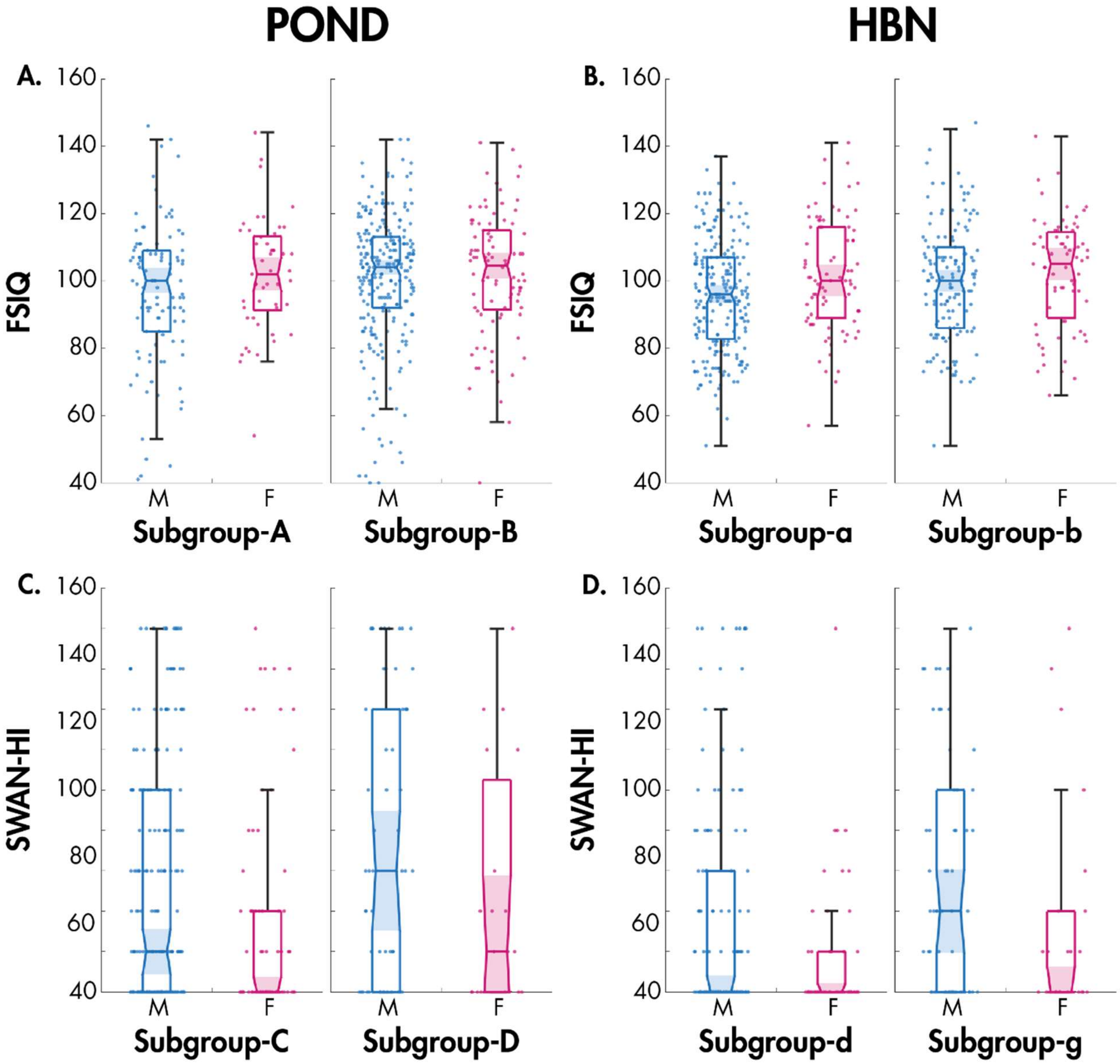

eFigure 4. Distributions of Intelligence (POND: A, HBN: B) and Hyperactivity/Impulsivity (POND: C, HBN: D), Separated for Males (M; blue) and Females (F; pink), for the Subgroups Showing Replicable Differences. POND: Province of Ontario Neurodevelopmental Network; HBN: Healthy Brain Network; FSIQ: full-scale intelligence quotient; SWAN-HI: Strengths and Weaknesses of ADHD-symptoms and Normal Behaviour hyperactivity/impulsivity subscale.

© 2023 Vandewouw MM et al. *JAMA Network Open.*

eTable 1. MRI Protocols for the T1-Weighted and Resting-State Data Acquired Using the 3 Scanners

**T1-weighted protocols**										
**Dataset**	**Scanner**	**Scan Type**	**N**	**Sequence**	**TR (ms)**	**TE (ms)**	**FA (°)**	**FOV (mm)**	**Voxel size (mm)**	**Scan time (min)**
POND	SK: 3T TimTrio	-	240	MPRAGE	2300	2.96	9	192×240×256	1	5.0
	QU: 3T TimTrio	-	112	MPRAGE	2300	3.14	9	192×240×256	0.8	6.2
	SK: 3T PrismaFIT	-	365	MPRAGE	1870	3.14	9	192×240×256	0.8	5.0
HBN	CBIC: 3T PrismaFIT	-	293	MPRAGE	2500	3.15	8	179×256×256	0.8	7.00
	RU: 3T TimTrio	-	477	MPRAGE	2500	3.15	8	179×256×256	0.8	7.00
	SI: 1.5T Avanto	-	188	MPRAGE	2730	1.64	7	176×256×256	1	6.53
**Resting-state protocols**										
**Dataset**	**Scanner**	**Scan Type**	**N**	**Sequence**	**TR (ms)**	**TE (ms)**	**FA (°)**	**FOV (mm)**	**Voxel size (mm)**	**Scan time (min)**
POND	SK: 3T TimTrio	Movies	240	EPI	2340	30	70	224×224×140	3.5	5.0
	QU: 3T TimTrio	Inscapes	112	EPI	2340	30	70	224×224×140	3.5	5.0
	SK: 3T PrismaFIT	Inscapes	365	EPI	1500	30	70	222×222×150	3	5.0
HBN	CBIC: 3T PrismaFIT	Fixation	293	EPI	800	30	31	202×202×144	2.4	2×5.1
	RU: 3T TimTrio	Fixation	477	EPI	800	30	31	202×202×144	2.4	2×5.1
	SI: 1.5T Avanto	Fixation	188	EPI	1450	40	55	195×195×135	2.5	10.3

POND: Province of Ontario Neurodevelopmental network; HBN: Healthy Brain Network; SK: Hospital for Sick Children, QU: Queen's University, CBIC: CitiGroup Cornell Brain Imaging Center, RU: Rutgers University, SI: Staten Island, TR: repetition time, TE: echo time, FA: flip angle, FOV: field of view

© 2023 Vandewouw MM et al. *JAMA Network Open.*

eTable 2. Normality Test Statistics for the Continuous Measures Describing the POND and HBN Sample Characteristics and the Clinical Behavioural Measures

Degrees of freedom indicate the sample size for each variable; participants with missing data were excluded from the statistical analysis on a case-by-case basis.

**Variable**	**Normality test statistics**	
	**POND**	**HBN**
Age	$W(551)=0.04$, $p=0.01$	$W(551)=0.05$, $p=1.05\times10^{-3}$
Head motion	$W(551)=0.17$, $p=2.72\times10^{-46}$	$W(551)=0.15$, $p=7.06\times10^{-35}$
FSIQ	$W(491)=0.08$, $p=3.43\times10^{-8}$	$W(518)=0.04$, $p=0.09$
SCQ	$W(501)=0.16$, $p=1.07\times10^{-33}$	$W(546)=0.13$, $p=5.58\times10^{-25}$
RBS-R	$W(513)=0.16$, $p=2.90\times10^{-34}$	$W(406)=0.21$, $p=1.74\times10^{-49}$
SWAN-I	$W(500)=0.18$, $p=8.51\times10^{-47}$	$W(540)=0.18$, $p=4.10\times10^{-48}$
SWAN-H/I	$W(500)=0.22$, $p=1.28\times10^{-65}$	$W(540)=0.28$, $p=3.73\times10^{-116}$
TOCS	$W(501)=0.10$, $p=5.44\times10^{-14}$	–
CBCL-OCS	–	$W(534)=0.16$, $p=3.39\times10^{-35}$

POND: Province of Ontario Neurodevelopmental network; HBN: Healthy Brain Network; FSIQ: full scale intelligence quotient; SCQ: Social Communication Questionnaire; RBS-R: Repetitive Behaviours Scale – Revised; SWAN-I: Strengths and Weaknesses of ADHD-symptoms and Normal Behaviour inattention subscale; SWAN-HI: Strengths and Weaknesses of ADHD-symptoms and Normal Behaviour hyperactivity/impulsivity subscale; TOCS: Toronto Obsessive-Compulsive Scale; CBCL-E: Child Behaviour Checklist obsessive-compulsive subscale

© 2023 Vandewouw MM et al. *JAMA Network Open.*

eTable 3. Race and Ethnicity Data for the POND and HBN Data Sets

		ADHD	ASD	OCD	TD
POND	Black	5	6	1	8
	East Asian	6	11	4	7
	Indigenous	10	4	1	7
	Latino	10	10	1	7
	Middle Eastern	1	5	3	1
	Other	12	14	3	6
	South Asian	7	6	4	4
	Southeast Asian	4	8	1	5
	White	95	98	30	76
HBN	Asian	6	4	1	8
	Black	66	10	0	6
	Hispanic	44	3	0	10
	Two or More (Mixed)	59	9	1	16
	Other	7	0	0	2
	White	170	30	8	49

ADHD: attention-deficit/hyperactivity disorder; ASD: autism spectrum disorder; OCD: obsessive-compulsive disorder; TD: typically developing; IQR: interquartile range; “Other” group: individuals who did not identify as one of the Canadian Institutes of Health Information or US census guideline categories

eTable 4. Descriptive Statistics of the Participant Demographics and Clinical Behavioural Measures Comparing the POND and HBN Data Sets, With Corresponding Statistics Identifying Significant (p<0.05) Differences Between the Data Sets, With the Directionality of the Difference Highlighted

Measure	Median (IQR)		Statistics			
	POND	HBN	Test statistic[a]	p-value	Effect size[b]	Directionality
N	551	551	–	–	–	–
Dx (ADHD:ASD:OCD:TD)	164:217:110:61	374:66:100:11	197.7	1.30×10^{-42}	0.42	ADHD: HBN > POND<br>ASD, OCD: POND > HBN
Age (years)	11.87 (5.25)	11.50 (4.98)	1.43×10^{5}	0.07	2.88×10^{-3}	–
Sex (M:F)	394:158	390:161	0.05	0.83	0.01	–
Race and ethnicity (Non-White:White)	158:228	252:257	6.50	0.01	0.09	Non-White: HBN > POND
Head motion (mm)	0.16 (0.13)	0.17 (0.13)	1.60×10^{5}	0.11	2.27×10^{-3}	–
FSIQ	103.00 (22.50)	99.00 (24.00)	1.15×10^{5}	0.01	7.44×10^{-3}	POND > HBN
SCQ	8.00 (15.00)	7.00 (6.00)	1.26×10^{5}	0.03	4.71×10^{-3}	POND > HBN
RBS-R	14.00 (25.00)	12.00 (37.00)	1.07×10^{5}	0.42	7.13×10^{-4}	–
SWAN-I	4.00 (7.00)	3.00 (6.00)	1.27×10^{5}	0.07	3.06×10^{-3}	–
SWAN-H/I	1.00 (5.00)	0.00 (3.00)	1.09×10^{5}	2.21×10^{-8}	0.03	POND > HBN

ADHD: attention-deficit/hyperactivity disorder; ASD: autism spectrum disorder; OCD: obsessive-compulsive disorder; TD: typically developing; IQR: interquartile range; SD: standard deviation; M: male; F: female; FD: framewise displacement; FSIQ: full scale intelligence quotient; SCQ: Social Communication Questionnaire; RBS-R: Repetitive Behaviours Scale – Revised; SWAN-I: Strengths and Weaknesses of ADHD-symptoms and Normal Behaviour inattention subscale; SWAN-HI: Strengths and Weaknesses of ADHD-symptoms and Normal Behaviour hyperactivity/impulsivity subscale; [a]Test statistic: Mann-Whitney *U*-statistic for non-normally distributed continuous variables, *t*-statistic for normally distributed continuous variables, and chi-squared X^2 for categorical variables; [b]Effect size: eta-squared (η^2) for continuous variables and Cramer's *V* for categorical variables

© 2023 Vandewouw MM et al. *JAMA Network Open.*

eTable 5. Descriptive Statistics of the Participant Demographics and Clinical Behavioural Measures for Each Leaf Cluster for Each Layer of the POND Dendrogram

	Median (IQR)									
**Dendrogram layer**	**2-cluster solution**		**3-cluster solution**		**4-cluster solution**		**5-cluster solution**		**6-cluster solution**	
**Leaf cluster**	**A**	**B**	**C**	**D**	**E**	**F**	**G**	**H**	**I**	**J**
**Dx (ADHD:ASD:OCD:TD)**	45:70:26:31	119:147:35:79	97:112:28:62	22:35:7:17	66:94:24:53	31:18:4:9	26:58:15:24	40:36:9:29	20:32:10:12	25:38:16:19
**Age (years)**	11.77 (5.51)	11.91 (5.15)	12.24 (5.02)	11.09 (4.69)	12.12 (5.38)	12.55 (4.53)	12.57 (4.92)	11.31 (5.10)	11.44 (5.48)	12.01 (5.67)
**Sex (M:F)**	118:54	276:104	216:83	60:21	171:66	45:17	89:34	82:32	47:27	71:27
**Primary caregiver education (Level 1:2:3:4:5)**	0:17:22:34:28	5:26:51:81:58	3:23:38:59:49	2:3:13:22:9	1:16:30:51:42	2:7:8:8:7	1:8:16:21:22	0:8:14:30:20	0:7:11:19:11	0:10:11:15:17
**Household income (Low:Medium:High)**	20:43:24	40:97:62	27:80:46	13:17:16	19:63:39	8:17:7	7:28:30	12:35:19	9:22:12	11:21:12
**Race and ethnicity (Non-White:White)**	49:66	109:162	84:125	25:37	72:95	12:30	36:47	36:48	24:27	25:39
**Scanner (SK-TT:QU-TT:SK-PF)**	91:60:21	196:125:59	153:101:45	43:24:14	125:78:34	28:23:11	64:47:12	61:31:22	41:25:8	50:35:13
**Head motion (mm)**	0.15 (0.16)	0.16 (0.13)	0.15 (0.14)	0.17 (0.10)	0.15 (0.14)	0.14 (0.10)	0.14 (0.08)	0.18 (0.19)	0.18 (0.16)	0.14 (0.13)
**FSIQ**	100.00 (22.00)	104.00 (22.00)	105.00 (20.25)	102.50 (27.50)	104.00 (21.00)	108.00 (18.75)	104.00 (26.00)	104.00 (15.75)	100.00 (22.00)	99.00 (23.00)
**SCQ**	9.00 (15.00)	6.50 (15.00)	6.00 (14.50)	7.50 (16.00)	6.00 (16.00)	6.00 (9.50)	10.00 (19.00)	5.00 (10.00)	11.00 (15.00)	9.00 (14.00)
**RBS-R**	17.00 (26.25)	13.00 (24.00)	11.00 (25.00)	17.00 (27.50)	13.00 (25.00)	10.50 (19.00)	14.00 (27.75)	9.00 (22.50)	17.00 (21.50)	15.00 (27.50)
**SWAN-I**	4.00 (7.00)	3.50 (7.00)	3.50 (7.00)	3.50 (6.00)	3.00 (7.00)	5.00 (6.00)	3.00 (6.75)	3.50 (7.00)	4.00 (7.00)	5.00 (7.00)
**SWAN-H/I**	2.00 (5.00)	1.00 (5.00)	1.00 (5.00)	2.50 (7.00)	1.00 (4.00)	2.00 (6.00)	1.00 (3.00)	1.00 (5.00)	2.00 (5.00)	2.00 (4.00)
**TOCS**	-5.00 (44.00)	-15.00 (51.00)	-17.00 (51.75)	-1.50 (46.00)	-16.00 (48.00)	-33.00 (48.75)	-12.50 (55.50)	-21.00 (45.00)	-5.00 (49.50)	-5.00 (40.50)

Dx: Diagnosis; ADHD: attention-deficit/hyperactivity disorder; ASD: autism spectrum disorder; OCD: obsessive-compulsive disorder; TD: typically developing; IQR: interquartile range; M: male; F: female; SK-TT: SickKids TimTrio; QU-TT: Queen's University TimTrio; SK-PF: SickKids PrismaFIT; FSIQ: full scale intelligence quotient; SCQ: Social Communication Questionnaire; RBS-R: Repetitive Behaviours Scale – Revised; SWAN-I: Strengths and Weaknesses of ADHD-symptoms and Normal Behaviour inattention subscale; SWAN-HI: Strengths and Weaknesses of ADHD-symptoms and Normal Behaviour hyperactivity/impulsivity subscale; TOCS: Toronto Obsessive-Compulsive Scale

© 2023 Vandewouw MM et al. *JAMA Network Open.*

eTable 6. Descriptive Statistics of the Participant Demographics and Clinical Behavioural Measures for Each Leaf Cluster for Each Layer of the HBN Dendrogram

	Median (IQR)									
**Dendrogram layer**	**2-cluster solution**		**3-cluster solution**		**4-cluster solution**		**5-cluster solution**		**6-cluster solution**	
**Leaf cluster**	**a**	**b**	**c**	**d**	**e**	**f**	**g**	**h**	**i**	**j**
**Dx (ADHD:ASD:OCD:TD)**	228:40:7:56	146:26:4:44	95:17:2:20	133:23:5:36	111:20:2:33	35:6:2:11	56:11:1:16	55:9:1:17	98:20:4:26	35:3:1:10
**Age (years)**	11.58 (5.09)	11.39 (4.86)	11.25 (4.92)	11.73 (5.26)	11.35 (4.87)	11.90 (4.90)	11.49 (4.82)	11.33 (4.81)	11.74 (4.91)	11.64 (5.60)
**Sex (M:F)**	242:89	148:72	103:31	139:58	115:51	33:21	59:25	56:26	101:47	38:11
**BSMSS**	51.25 (20.25)	50.00 (16.50)	53.00 (14.12)	50.00 (21.25)	50.00 (16.50)	50.75 (15.50)	49.75 (18.50)	51.50 (16.00)	50.00 (19.00)	46.25 (29.00)
**Race and ethnicity (Non-White:White)**	155:150	97:107	59:65	96:85	80:79	17:28	43:40	37:39	65:71	31:14
**Scanner (CBIC:RU:SI)**	124:156:51	79:104:37	48:71:15	76:85:36	61:78:27	18:26:10	29:39:16	32:39:11	56:64:28	20:21:8
**Mean head motion (mm)**	0.16 (0.11)	0.17 (0.14)	0.15 (0.10)	0.17 (0.12)	0.17 (0.15)	0.17 (0.11)	0.17 (0.15)	0.18 (0.18)	0.17 (0.12)	0.18 (0.11)
**Mean FSIQ (SD)**	97.08 (16.55)	100.63 (16.98)	96.44 (16.13)	97.51 (16.86)	101.57 (17.43)	97.51 (15.14)	101.28 (18.65)	101.86 (16.20)	98.75 (17.53)	93.71 (14.10)
**SCQ**	7.00 (7.00)	7.00 (6.00)	7.00 (6.00)	7.00 (7.00)	7.00 (7.00)	7.00 (5.50)	7.00 (7.00)	6.00 (6.00)	7.00 (7.00)	7.00 (5.25)
**RBS-R**	11.00 (37.00)	12.00 (44.00)	15.00 (40.00)	10.00 (29.25)	13.00 (44.00)	8.00 (38.50)	24.00 (54.50)	8.00 (27.50)	7.00 (27.75)	11.50 (35.00)
**SWAN-I**	3.00 (6.00)	3.00 (6.00)	3.00 (5.00)	3.00 (6.00)	3.00 (6.00)	3.00 (5.00)	4.00 (7.00)	2.00 (4.25)	2.50 (6.00)	3.00 (5.00)
**SWAN-H/I**	0.00 (2.00)	1.00 (3.00)	1.00 (3.00)	0.00 (2.00)	1.00 (3.00)	0.00 (2.00)	1.00 (4.00)	0.00 (2.00)	0.00 (2.00)	0.00 (3.00)
**CBCL-OCS**	3.00 (2.00)	3.00 (3.00)	3.00 (3.00)	2.00 (2.00)	3.00 (3.00)	3.00 (3.00)	3.00 (4.00)	2.00 (2.25)	2.50 (2.00)	2.00 (2.00)

Dx: Diagnosis; ADHD: attention-deficit/hyperactivity disorder; ASD: autism spectrum disorder; OCD: obsessive-compulsive disorder; TD: typically developing; IQR: interquartile range; SD: standard deviation; M: male; F: female; CBIC: CitiGroup Cornell Brain Imaging Center; RU: Rutgers University; SI: Staten Island; FSIQ: full scale intelligence quotient; SCQ: Social Communication Questionnaire; RBS-R: Repetitive Behaviours Scale – Revised; SWAN-I: Strengths and Weaknesses of ADHD-symptoms and Normal Behaviour inattention subscale; SWAN-HI: Strengths and Weaknesses of ADHD-symptoms and Normal Behaviour hyperactivity/impulsivity subscale; CBCL-OCS: Child Behaviour Checklist obsessive-compulsive subscale

eTable 7. Statistical Details of the Mann Whitney U and *t* Tests and χ² Tests Examining Differences in Sample Characteristics Between the Leaf Clusters in Each Layer of the POND Dendrogram

	Test statistic[a]					*p*-value					Effect size[b]				
**Dendrogram layer**	**2**	**3**	**4**	**5**	**6**	**2**	**3**	**4**	**5**	**6**	**2**	**3**	**4**	**5**	**6**
**Dx**	5.37	1.15	11.08	9.76	0.70	0.15	0.76	0.01	0.02	0.87	0.10	0.06	0.19	0.20	0.06
**Age**	3.17	1.08	0.76	0.58	0.38	0.58	0.15	0.64	0.02	0.49	0.00	0.01	0.00	0.02	0.00
**Sex**	0.94	0.11	0.00	0.01	1.56	0.33	0.74	0.95	0.94	0.21	0.04	0.02	0.00	0.00	0.10
**Caregiver education**	0.01	0.26	4.67	0.02	0.06	0.92	0.61	0.03	0.88	0.80	0.00	0.00	0.03	0.00	0.00
**Household income**	0.50	0.17	2.11	1.12	0.09	0.48	0.68	0.15	0.29	0.76	0.00	0.00	0.01	0.01	0.00
**Race and ethnicity**	0.19	0.00	2.95	0.00	0.74	0.66	0.99	0.09	0.95	0.39	0.02	0.00	0.12	0.01	0.08
**Scanner**	1.08	0.58	1.18	5.96	0.41	0.58	0.75	0.55	0.05	0.82	0.04	0.04	0.06	0.16	0.05
**Head motion**	3.18	1.29	0.72	0.91	0.30	0.60	0.37	0.84	7.97×10^{-5}	0.05	0.00	0.00	0.00	0.07	0.02
**FSIQ**	2.86	0.90	0.66	0.58	0.28	0.04	0.35	0.03	0.78	0.89	0.01	0.00	0.02	0.00	0.00
**SCQ**	2.49	1.05	0.57	0.45	0.30	0.17	0.32	0.47	3.05×10^{-3}	0.90	0.00	0.00	0.00	0.04	0.00
**RBS-R**	2.70	1.18	0.56	0.53	0.29	0.41	0.06	0.44	0.06	0.42	0.00	0.01	0.00	0.02	0.00
**SWAN-I**	2.51	0.99	0.66	0.62	0.29	0.29	0.88	0.22	0.42	0.94	0.00	0.00	0.01	0.00	0.00
**SWAN-H/I**	2.59	1.19	0.65	0.62	0.26	0.59	0.01	0.28	0.46	0.27	0.00	0.02	0.00	0.00	0.01
**TOCS**	2.46	1.20	0.51	0.51	0.30	0.13	0.01	0.21	0.10	0.93	0.00	0.02	0.01	0.01	0.00

Dx: Diagnosis; FSIQ: full scale intelligence quotient; SCQ: Social Communication Questionnaire; RBS-R: Repetitive Behaviours Scale – Revised; SWAN-I: Strengths and Weaknesses of ADHD-symptoms and Normal Behaviour inattention subscale; SWAN-HI: Strengths and Weaknesses of ADHD-symptoms and Normal Behaviour hyperactivity/impulsivity subscale; TOCS: Toronto Obsessive-Compulsive Scale; [a]Test statistic: Mann-Whitney *U*-statistic ($\times10^4$) for non-normally distributed continuous variables, *t*-statistic for normally distributed continuous variables, and chi-squared X^2 for categorical variables; [b]Effect size: eta-squared (η^2) for continuous variables and Cramer's *V* for categorical variables

© 2023 Vandewouw MM et al. *JAMA Network Open.*

eTable 8. Statistical Details of the Mann Whitney U and *t* Tests and χ² Tests Examining Differences in Sample Characteristics Between the Leaf Clusters in Each Layer of the HBN Dendrogram

	Test statistic[a]					*p*-value					Effect size[b]				
**Dendrogram layer**	**2**	**3**	**4**	**5**	**6**	**2**	**3**	**4**	**5**	**6**	**2**	**3**	**4**	**5**	**6**
**Dx**	0.88	1.14	1.46	0.22	2.10	0.83	0.77	0.69	0.98	0.55	0.04	0.06	0.08	0.04	0.10
**Age**	3.59	1.37	0.52	0.35	0.34	0.80	0.53	0.07	0.94	0.62	0.00	0.00	0.02	0.00	0.00
**Sex**	2.18	1.61	1.23	0.07	1.54	0.14	0.20	0.27	0.79	0.22	0.06	0.07	0.07	0.02	0.09
**BSMSS**	3.41	1.15	4.21	0.39	0.30	0.60	0.14	0.88	0.10	0.15	0.00	0.01	0.00	0.02	0.01
**Race and ethnicity**	0.52	0.88	2.21	0.15	6.04	0.47	0.35	0.14	0.69	0.01	0.03	0.05	0.10	0.03	0.18
**Scanner**	0.25	4.39	0.27	1.05	0.22	0.88	0.11	0.88	0.59	0.90	0.02	0.12	0.03	0.08	0.03
**Head motion**	3.89	1.44	0.43	0.37	0.41	0.18	0.15	0.59	0.35	0.18	0.00	0.01	0.00	0.01	0.01
**FSIQ**	2.37	0.56	1.47	0.21	1.75	0.02	0.58	0.14	0.83	0.08	0.01	0.00	0.01	0.00	0.02
**SCQ**	3.37	1.34	0.42	0.33	0.37	0.28	0.65	0.75	0.82	0.90	0.00	0.00	0.00	0.00	0.00
**RBS-R**	2.04	0.69	0.22	0.15	0.23	0.44	0.19	0.85	0.15	0.16	0.00	0.01	0.00	0.01	0.01
**SWAN-I**	3.48	1.19	0.40	0.25	0.36	0.92	0.32	0.74	4.76×10^{-3}	0.62	0.00	0.00	0.00	0.05	0.00
**SWAN-H/I**	3.68	1.13	0.38	0.27	0.38	0.27	0.06	0.35	0.02	0.18	0.00	0.01	0.00	0.03	0.01
**CBCL-OCS**	3.31	1.19	0.39	0.31	0.33	0.50	0.59	0.65	0.33	0.68	0.00	0.00	0.00	0.01	0.00

Dx: Diagnosis; FSIQ: full scale intelligence quotient; SCQ: Social Communication Questionnaire; RBS-R: Repetitive Behaviours Scale – Revised; SWAN-I: Strengths and Weaknesses of ADHD-symptoms and Normal Behaviour inattention subscale; SWAN-HI: Strengths and Weaknesses of ADHD-symptoms and Normal Behaviour hyperactivity/impulsivity subscale; CBCL-E: Child Behaviour Checklist obsessive-compulsive subscale; [a]Test statistic: Mann-Whitney *U*-statistic ($\times10^4$) for non-normally distributed continuous variables, *t*-statistic for normally distributed continuous variables, and chi-squared X^2 for categorical variables; [b]Effect size: eta-squared (η^2) for continuous variables and Cramer's *V* for categorical variables

© 2023 Vandewouw MM et al. *JAMA Network Open.*

eTable 9. Descriptive Statistics of the Network-Averaged Measures of Segregation and Integration for Each Leaf Cluster for Each Layer of the POND Dendrogram

Median segregation ×10 (IQR)									
**Dendrogram layer**	**Leaf cluster**	**VIS**	**Mean SMOT (SD)**	**DAN**	**SAL/VAN**	**LIM**	**FP-CONT**	**Mean DMN (SD)**	**SC**
**2-cluster solution**	**A**	1.52 (0.59)	2.08 (0.54)	1.27 (0.39)	1.01 (0.36)	1.05 (0.47)	1.06 (0.32)	1.10 (0.34)	0.90 (0.30)
	**B**	2.20 (0.73)	2.75 (0.59)	1.80 (0.51)	1.64 (0.63)	1.37 (0.53)	1.66 (0.56)	1.43 (0.37)	1.44 (0.53)
**3-cluster solution**	**C**	2.10 (0.69)	2.67 (0.47)	1.74 (0.40)	1.54 (0.47)	1.33 (0.50)	1.56 (0.40)	1.38 (0.35)	1.31 (0.45)
	**D**	2.62 (0.57)	3.16 (0.38)	2.28 (0.47)	2.17 (0.45)	1.59 (0.53)	2.24 (0.40)	1.61 (0.31)	1.96 (0.53)
**4-cluster solution**	**E**	2.00 (0.57)	2.60 (0.46)	1.69 (0.34)	1.46 (0.42)	1.28 (0.51)	1.51 (0.32)	1.32 (0.29)	1.24 (0.39)
	**F**	2.50 (0.53)	2.80 (0.33)	1.97 (0.37)	1.88 (0.46)	1.36 (0.38)	1.86 (0.43)	1.63 (0.26)	1.62 (0.36)
**5-cluster solution**	**G**	2.07 (0.71)	2.58 (0.44)	1.68 (0.31)	1.40 (0.44)	1.16 (0.37)	1.48 (0.31)	1.26 (0.24)	1.19 (0.33)
	**H**	1.95 (0.48)	2.65 (0.51)	1.69 (0.38)	1.50 (0.37)	1.48 (0.55)	1.53 (0.34)	1.41 (0.33)	1.37 (0.37)
**6-cluster solution**	**I**	1.38 (0.43)	1.93 (0.56)	1.11 (0.42)	0.86 (0.33)	1.04 (0.48)	0.97 (0.24)	1.01 (0.32)	0.79 (0.24)
	**J**	1.73 (0.52)	2.21 (0.53)	1.36 (0.34)	1.12 (0.27)	1.06 (0.44)	1.15 (0.32)	1.15 (0.29)	1.01 (0.28)
**Median integration ×10^3 (IQR)**									
**Dendrogram layer**	**Leaf cluster**	**VIS**	**SMOT**	**DAN**	**SAL/VAN**	**LIM**	**FP-CONT**	**DMN**	**SC**
**2-cluster solution**	**A**	7.51 (2.41)	5.53 (2.09)	7.75 (3.60)	7.26 (3.25)	2.73 (1.52)	8.12 (4.31)	7.34 (2.70)	9.28 (4.57)
	**B**	7.76 (2.36)	8.86 (3.37)	9.20 (3.26)	7.04 (2.63)	4.06 (2.01)	6.19 (2.64)	10.34 (3.17)	5.64 (2.66)
**3-cluster solution**	**C**	7.92 (2.34)	8.27 (2.55)	9.00 (3.23)	7.23 (2.78)	4.13 (1.98)	6.64 (2.46)	9.60 (2.51)	6.15 (2.47)
	**D**	6.69 (2.76)	11.81 (3.97)	10.24 (4.06)	6.26 (2.37)	3.59 (2.20)	4.27 (1.70)	12.68 (3.07)	4.25 (1.40)
**4-cluster solution**	**E**	7.92 (2.40)	7.87 (2.38)	9.08 (2.90)	7.25 (2.67)	4.05 (1.72)	6.74 (2.39)	9.40 (2.86)	6.40 (2.89)
	**F**	7.91 (1.83)	10.74 (3.17)	8.47 (3.28)	7.14 (2.92)	4.76 (2.78)	6.24 (1.98)	10.37 (2.01)	5.58 (1.59)
**5-cluster solution**	**G**	7.88 (2.20)	7.98 (2.14)	9.79 (2.79)	7.32 (2.89)	3.71 (1.47)	6.54 (2.48)	10.07 (2.61)	5.43 (2.62)
	**H**	7.96 (2.59)	7.72 (2.70)	8.52 (2.66)	7.01 (2.53)	4.35 (2.49)	6.93 (2.43)	8.80 (2.70)	7.05 (3.20)
**6-cluster solution**	**I**	7.31 (2.58)	4.69 (1.62)	7.80 (3.73)	7.13 (4.26)	2.22 (1.42)	8.87 (4.86)	6.91 (2.38)	11.02 (5.02)
	**J**	7.64 (2.49)	6.11 (1.82)	7.67 (3.34)	7.27 (2.95)	3.00 (1.41)	7.88 (3.64)	7.78 (2.69)	8.69 (3.29)

IQR: interquartile range; SD: standard deviation; VIS: visual network; SMOT: somatomotor network; DAN: dorsal attention network; SAL/VAN: salience/ventral attention network; LIM: limbic network; FP-CONT: frontoparietal control network; DMN: default mode network; SC: subcortical regions

© 2023 Vandewouw MM et al. *JAMA Network Open.*

eTable 10. Descriptive Statistics of the Network-Averaged Measures of Segregation and Integration for Each Leaf Cluster for Each Layer of the HBN Dendrogram

Median segregation ×10 (IQR)									
**Dendrogram layer**	**Leaf cluster**	**Mean VIS (SD)**	**Mean SMOT (SD)**	**Mean DAN (SD)**	**SAL/VAN**	**LIM**	**FP-CONT**	**Mean DMN (SD)**	**SC**
**2-cluster solution**	**a**	1.85 (0.76)	2.30 (0.69)	1.42 (0.51)	1.09 (0.51)	0.99 (0.40)	1.20 (0.49)	1.14 (0.33)	0.85 (0.30)
	**b**	2.53 (0.57)	2.98 (0.49)	2.12 (0.44)	1.83 (0.50)	1.44 (0.60)	1.98 (0.52)	1.53 (0.30)	1.38 (0.63)
**3-cluster solution**	**c**	2.06 (0.53)	2.47 (0.40)	1.62 (0.40)	1.26 (0.34)	1.08 (0.40)	1.41 (0.35)	1.26 (0.25)	0.98 (0.25)
	**d**	1.63 (0.66)	2.06 (0.73)	1.27 (0.46)	0.91 (0.47)	0.91 (0.40)	1.02 (0.37)	1.05 (0.30)	0.77 (0.24)
**4-cluster solution**	**e**	2.49 (0.56)	2.87 (0.47)	2.08 (0.47)	1.76 (0.48)	1.37 (0.59)	1.87 (0.41)	1.51 (0.32)	1.27 (0.42)
	**f**	2.65 (0.67)	3.18 (0.50)	2.28 (0.59)	2.02 (0.45)	1.59 (0.41)	2.30 (0.34)	1.58 (0.22)	1.88 (0.54)
**5-cluster solution**	**g**	2.34 (0.52)	2.74 (0.54)	1.92 (0.39)	1.60 (0.40)	1.19 (0.52)	1.77 (0.34)	1.37 (0.21)	1.12 (0.32)
	**h**	2.65 (0.54)	2.98 (0.49)	2.22 (0.35)	1.94 (0.43)	1.59 (0.59)	1.95 (0.42)	1.65 (0.26)	1.44 (0.48)
**6-cluster solution**	**i**	1.55 (0.63)	1.92 (0.60)	1.21 (0.44)	0.84 (0.33)	0.91 (0.37)	1.00 (0.28)	1.04 (0.27)	0.75 (0.24)
	**j**	1.80 (0.98)	2.81 (0.52)	1.58 (0.78)	1.26 (0.60)	1.01 (0.48)	1.27 (0.77)	1.12 (0.41)	0.81 (0.28)
**Median integration ×10^3 (IQR)**									
**Dendrogram layer**	**Leaf cluster**	**VIS**	**SMOT**	**DAN**	**SAL/VAN**	**LIM**	**FP-CONT**	**DMN**	**SC**
**2-cluster solution**	**a**	7.88 (2.84)	6.34 (2.80)	8.20 (3.67)	7.07 (3.20)	2.69 (1.84)	7.93 (3.87)	8.02 (2.72)	8.52 (5.27)
	**b**	7.27 (2.96)	10.11 (3.07)	9.38 (3.52)	7.25 (2.66)	4.25 (2.64)	5.28 (2.27)	11.60 (3.12)	4.69 (1.95)
**3-cluster solution**	**c**	8.42 (2.69)	6.94 (2.03)	8.65 (3.28)	7.44 (2.63)	3.55 (2.05)	7.69 (3.16)	9.21 (2.70)	6.33 (2.79)
	**d**	7.53 (2.86)	5.71 (2.83)	7.85 (3.74)	6.70 (3.12)	2.17 (1.27)	8.48 (4.42)	7.33 (2.30)	10.41 (5.34)
**4-cluster solution**	**e**	7.96 (2.69)	10.04 (3.34)	9.33 (3.61)	7.15 (2.61)	4.36 (2.44)	5.65 (2.11)	10.84 (3.00)	4.95 (2.05)
	**f**	5.98 (1.85)	10.19 (1.61)	9.46 (2.94)	7.86 (2.62)	3.71 (2.45)	4.03 (1.68)	13.15 (2.07)	3.90 (1.46)
**5-cluster solution**	**g**	8.41 (2.34)	9.35 (2.94)	10.75 (4.04)	7.09 (3.10)	4.04 (2.13)	5.14 (1.61)	11.39 (2.61)	5.18 (1.87)
	**h**	7.22 (2.78)	10.38 (4.05)	8.58 (3.05)	7.29 (2.54)	5.21 (3.17)	6.30 (2.10)	10.36 (2.67)	4.94 (2.09)
**6-cluster solution**	**i**	7.60 (2.88)	4.86 (2.20)	8.19 (3.56)	7.16 (3.23)	2.15 (1.28)	9.00 (4.22)	7.37 (2.18)	9.51 (4.62)
	**j**	7.24 (2.66)	7.82 (2.83)	6.95 (3.65)	5.23 (2.14)	2.23 (1.24)	5.46 (3.97)	6.89 (2.88)	14.10 (5.79)

IQR: interquartile range; SD: standard deviation; VIS: visual network; SMOT: somatomotor network; DAN: dorsal attention network; SAL/VAN: salience/ventral attention network; LIM: limbic network; FP-CONT: frontoparietal control network; DMN: default mode network; SC: subcortical regions

eTable 11. Statistical Details of the Tests Examining Differences in the Network-Averaged Measures of Segregation and Integration Between the Leaf Clusters in Each Layer of the POND Dendrogram

		**Test statistic**					***p*-value**					**Effect size (η^2)**				
**Dendrogram layer**		**2**	**3**	**4**	**5**	**6**	**2**	**3**	**4**	**5**	**6**	**2**	**3**	**4**	**5**	**6**
**Segregation**	**VIS**	0.62	0.50	0.49	0.08	0.33	4.99×10^{-17}	6.89×10^{-9}	4.91×10^{-9}	1.00	8.33×10^{-5}	0.28	0.16	0.17	0.01	0.15
	**SMOT**	0.68	0.48	0.19	0.07	0.35	1.30×10^{-21}	6.36×10^{-14}	0.01	1.00	8.50×10^{-6}	0.37	0.23	0.04	0.01	0.17
	**DAN**	0.60	0.55	0.34	0.04	0.26	2.46×10^{-23}	2.55×10^{-19}	8.43×10^{-10}	1.00	2.14×10^{-6}	0.40	0.29	0.18	0.00	0.19
	**SAL/VAN**	0.69	0.56	0.44	0.11	0.23	5.72×10^{-22}	1.17×10^{-14}	1.93×10^{-11}	0.20	9.04×10^{-7}	0.46	0.26	0.22	0.03	0.21
	**LIM**	0.29	0.24	0.07	0.31	0.08	1.96×10^{-10}	9.84×10^{-6}	1.00	5.96×10^{-8}	1.00	0.13	0.07	0.01	0.18	0.01
	**FP-CONT**	0.64	0.62	0.34	0.07	0.18	3.97×10^{-22}	9.08×10^{-17}	8.04×10^{-10}	0.83	1.92×10^{-5}	0.47	0.37	0.18	0.01	0.15
	**DMN**	0.32	0.21	0.26	0.18	0.13	2.34×10^{-17}	8.81×10^{-8}	6.55×10^{-10}	4.08×10^{-7}	4.93×10^{-3}	0.27	0.11	0.19	0.16	0.08
	**SC**	0.55	0.60	0.28	0.15	0.25	1.95×10^{-20}	1.84×10^{-16}	3.99×10^{-7}	1.13×10^{-3}	3.49×10^{-8}	0.40	0.32	0.15	0.08	0.28
**Integration**	**VIS**	0.05	1.35	0.27	0.14	0.48	1.00	1.46×10^{-6}	1.00	1.00	1.00	0.00	0.08	0.00	0.00	0.01
	**SMOT**	3.47	3.22	2.59	0.39	1.30	3.46×10^{-19}	1.23×10^{-12}	2.95×10^{-11}	1.00	8.63×10^{-6}	0.39	0.21	0.22	0.02	0.19
	**DAN**	1.29	0.79	0.66	1.58	0.14	3.44×10^{-6}	0.24	1.00	1.18×10^{-4}	1.00	0.06	0.02	0.01	0.12	0.00
	**SAL/VAN**	0.33	0.90	0.44	0.36	0.04	1.00	0.02	1.00	1.00	1.00	0.00	0.03	0.00	0.00	0.00
	**LIM**	1.42	0.49	0.70	0.92	0.61	1.14×10^{-10}	0.36	0.08	3.67×10^{-4}	0.04	0.18	0.01	0.02	0.06	0.07
	**FP-CONT**	2.26	2.38	0.69	0.37	0.53	8.17×10^{-15}	2.25×10^{-11}	0.26	1.00	1.00	0.15	0.26	0.02	0.01	0.01
	**DMN**	2.85	2.97	0.95	1.24	0.70	1.01×10^{-16}	7.32×10^{-16}	0.02	4.39×10^{-4}	0.33	0.28	0.26	0.05	0.09	0.03
	**SC**	3.71	2.35	1.16	2.36	1.73	1.12×10^{-18}	1.38×10^{-10}	0.02	6.03×10^{-8}	0.02	0.27	0.22	0.03	0.20	0.07

VIS: visual network; SMOT: somatomotor network; DAN: dorsal attention network; SAL/VAN: salience/ventral attention network; LIM: limbic network; FP-CONT: frontoparietal control network; DMN: default mode network; SC: subcortical regions

© 2023 Vandewouw MM et al. *JAMA Network Open.*

eTable 12. Statistical Details of the Tests Examining Differences in the Network-Averaged Measures of Segregation and Integration Between the Leaf Clusters in Each Layer of the HBN Dendrogram

		Test statistic[a]					*p*-value					Effect size[b]				
**Dendrogram layer**		**2**	**3**	**4**	**5**	**6**	**2**	**3**	**4**	**5**	**6**	**2**	**3**	**4**	**5**	**6**
**Segregation**	**VIS**	0.68	0.44	0.17	0.37	0.31	5.42×10^{-18}	7.07×10^{-9}	0.39	1.41×10^{-5}	0.01	0.30	0.16	0.02	0.16	0.06
	**SMOT**	0.70	0.33	0.38	0.20	0.86	1.22×10^{-25}	8.00×10^{-7}	2.92×10^{-7}	0.01	5.35×10^{-14}	0.35	0.10	0.18	0.08	0.47
	**DAN**	0.70	0.30	0.29	0.32	0.43	7.10×10^{-26}	9.15×10^{-8}	4.64×10^{-5}	2.20×10^{-6}	2.63×10^{-8}	0.46	0.15	0.11	0.22	0.23
	**SAL/VAN**	0.72	0.30	0.31	0.30	0.48	7.28×10^{-27}	4.98×10^{-9}	3.96×10^{-7}	1.57×10^{-6}	4.57×10^{-9}	0.49	0.20	0.12	0.19	0.25
	**LIM**	0.42	0.14	0.20	0.39	0.05	5.41×10^{-15}	3.04×10^{-3}	0.04	1.38×10^{-6}	1.00	0.24	0.06	0.06	0.20	0.00
	**FP-CONT**	0.77	0.31	0.44	0.15	0.33	8.36×10^{-26}	7.84×10^{-10}	7.95×10^{-11}	0.03	2.97×10^{-8}	0.56	0.25	0.28	0.07	0.10
	**DMN**	0.39	0.20	0.08	0.29	0.13	1.73×10^{-19}	5.44×10^{-9}	0.35	6.48×10^{-11}	0.02	0.39	0.16	0.02	0.39	0.06
	**SC**	0.59	0.20	0.55	0.30	0.07	2.08×10^{-25}	8.67×10^{-12}	4.97×10^{-12}	2.51×10^{-6}	0.50	0.48	0.21	0.29	0.21	0.01
**Integration**	**VIS**	0.72	0.83	1.75	0.61	0.64	0.01	0.03	2.48×10^{-5}	1.00	1.00	0.02	0.04	0.14	0.06	0.01
	**SMOT**	3.69	1.39	0.16	1.14	3.19	3.40×10^{-20}	2.60×10^{-7}	1.00	4.85×10^{-2}	5.92×10^{-11}	0.42	0.13	0.00	0.04	0.30
	**DAN**	1.31	0.63	0.08	2.10	0.96	7.90×10^{-7}	0.50	1.00	3.81×10^{-5}	0.47	0.06	0.02	0.00	0.14	0.03
	**SAL/VAN**	0.19	0.85	0.28	0.40	1.92	1.00	0.05	1.00	1.00	1.66×10^{-4}	0.00	0.04	0.01	0.01	0.13
	**LIM**	1.67	1.36	0.78	1.70	0.27	1.12×10^{-10}	7.17×10^{-8}	0.69	6.18×10^{-4}	1.00	0.18	0.24	0.03	0.10	0.00
	**FP-CONT**	2.56	0.28	1.74	0.92	3.23	1.21×10^{-14}	1.00	3.31×10^{-8}	0.06	1.11×10^{-8}	0.23	0.00	0.19	0.08	0.22
	**DMN**	3.42	1.91	2.23	1.19	0.24	1.76×10^{-20}	1.80×10^{-8}	2.03×10^{-7}	4.64×10^{-3}	1.00	0.37	0.18	0.21	0.08	0.00
	**SC**	4.16	3.98	1.27	0.07	4.00	1.30×10^{-18}	1.06×10^{-12}	6.19×10^{-4}	1.00	1.92×10^{-7}	0.38	0.29	0.12	0.00	0.17

VIS: visual network; SMOT: somatomotor network; DAN: dorsal attention network; SAL/VAN: salience/ventral attention network; LIM: limbic network; FP-CONT: frontoparietal control network; DMN: default mode network; SC: subcortical region

© 2023 Vandewouw MM et al. *JAMA Network Open.*

eTable 13. Statistical Details of the Tests Examining Differences in the Network-Averaged Measures of Segregation and Integration Between the Leaf Clusters From the HBN Dendrogram That Differed in Hyperactivity/Impulsivity Problems (Subgroup d and g)

		Subgroup-d	Subgroup-g	Statistics		
				Test statistic[a]	*p*-value	Effect size[b]
**Median segregation (IQR)**	**Mean VIS (SD)**	1.67 (0.54)	2.30 (0.41)	0.63	3.74×10^{-9}	0.25
	**Mean SMOT (SD)**	2.14 (0.55)	2.78 (0.36)	0.64	1.62×10^{-10}	0.26
	**Mean DAN (SD)**	1.31 (0.39)	1.90 (0.30)	0.60	3.33×10^{-17}	0.36
	**SAL/VAN**	0.91 (0.47)	1.60 (0.40)	0.62	9.43×10^{-14}	0.58
	**LIM**	0.91 (0.40)	1.19 (0.52)	0.24	2.11×10^{-5}	0.12
	**FP-CONT**	1.02 (0.37)	1.77 (0.34)	0.72	1.51×10^{-15}	0.70
	**Mean DMN (SD)**	1.07 (0.25)	1.38 (0.18)	0.31	1.11×10^{-13}	0.28
	**SC**	0.77 (0.24)	1.12 (0.32)	0.38	3.32×10^{-14}	0.54
**Median integration (IQR)**	**VIS**	7.53 (2.86)	8.41 (2.34)	0.35	1.00	0.03
	**SMOT**	5.71 (2.83)	9.35 (2.94)	3.65	1.46×10^{-12}	0.54
	**DAN**	7.85 (3.74)	10.75 (4.04)	2.58	4.10×10^{-8}	0.25
	**SAL/VAN**	6.70 (3.12)	7.09 (3.10)	0.27	1.00	0.01
	**LIM**	2.17 (1.27)	4.04 (2.13)	1.58	1.49×10^{-8}	0.39
	**FP-CONT**	8.48 (4.42)	5.14 (1.61)	2.70	2.02×10^{-9}	0.26
	**DMN**	7.33 (2.30)	11.39 (2.61)	4.23	8.85×10^{-18}	0.66
	**SC**	10.41 (5.34)	5.18 (1.87)	5.42	2.60×10^{-11}	0.61

VIS: visual network; SMOT: somatomotor network; DAN: dorsal attention network; SAL/VAN: salience/ventral attention network; LIM: limbic network; FP-CONT: frontoparietal control network; DMN: default mode network; SC: subcortical regions; [a]Test statistic: Mann-Whitney *U*-statistic for non-normally distributed continuous variables, and *t*-statistic for normally distributed continuous variables; [b]Effect size: eta-squared (η^2) for continuous variables

© 2023 Vandewouw MM et al. *JAMA Network Open.*

eReferences

1. Lord, C., Rutter, M., DiLavore, P. C., Risi, S., Gotham, K., & Bishop S. *Autism Diagnostic Observation Schedule, Second Edition (ADOS-2) Manual (Part I): Modules 1–4*. Western Psychological Services; 2012.

2. Lord C, Rutter M, Le Couteur A. Autism Diagnostic Interview-Revised: A revised version of a diagnostic interview for caregivers of individuals with possible pervasive developmental disorders. *J Autism Dev Disord*. 1994;24(5):659-685. doi:10.1007/bf02172145

3. Kaufman J, Birmaher B, Brent D, et al. Schedule for Affective Disorders and Schizophrenia for School-Age Children-Present and Lifetime Version (K-SADS-PL): Initial Reliability and Validity Data. *J Am Acad Child Adolesc Psychiatry*. 1997;36(7):980-988. doi:10.1097/00004583-199707000-00021

4. Ickowicz A, Schachar RJ, Sugarman R, Chen SX, Millette C, Cook L. The Parent Interview for Child Symptoms: A Situation-Specific Clinical Research Interview for Attention-Deficit Hyperactivity and Related Disorders. *The Canadian Journal of Psychiatry*. 2006;51(5):325-328. doi:10.1177/070674370605100508

5. *Diagnostic and Statistical Manual of Mental Disorders (DSM-5®)*. American Psychiatric Publishing; 2013.

6. Wechsler D. *Weschler Intelligence Scale for Children*. 4th ed. The Psychological Corporation; 2003.

7. Wechsler D. *Wechsler Intelligence Scale for Children*. 5th ed. Pearson; 2014.

8. Wechsler D. *Wechsler Abbreviated Scales of Intelligence*. The Psychological Corporation; 1999.

9. Wechsler D. *Wechsler Abbreviated Scale of Intelligence*. 2nd ed. NCS Pearson; 2011.

10. Wechsler D. *Wechsler Preschool and Primary Scales of Intelligence*. 3rd ed. The Psychological Corporation; 2002.

11. Roid GH. *The Stanford-Binet Intelligence Scales*. 5th ed. Riverside Publishing; 2003.

12. Rutter M, Bailey A, Lord C. *The Social Communication Questionnaire*. Western Psychological Services; 2003.

13. Bodfish JW, Symons FJ, Parker DE, Lewis MH. Varieties of repetitive behavior in autism: Comparisons to mental retardation. *J Autism Dev Disord*. 2000;30(3):237-243. doi:10.1023/A:1005596502855

14. Swanson JM, Schuck S, Porter MM, et al. Categorical and Dimensional Definitions and Evaluations of Symptoms of ADHD: History of the SNAP and the

© 2023 Vandewouw MM et al. *JAMA Network Open.*

SWAN Rating Scales. *Int J Educ Psychol Assess*. 2012;10(1):51-70. http://www.ncbi.nlm.nih.gov/pubmed/26504617%0Ahttp://www.pubmedcentral.nih.gov/articlerender.fcgi?artid=PMC4618695

15. Nelson EC, Hanna GL, Hudziak JJ, Botteron KN, Heath AC, Todd RD. Obsessive-compulsive scale of the child behavior checklist: specificity, sensitivity, and predictive power. *Pediatrics*. 2001;108(1). doi:10.1542/PEDS.108.1.E14

16. Park LS, Burton CL, Dupuis A, et al. The Toronto Obsessive-Compulsive Scale: Psychometrics of a Dimensional Measure of Obsessive-Compulsive Traits. *J Am Acad Child Adolesc Psychiatry*. 2016;55(4):310-318.e4. doi:10.1016/j.jaac.2016.01.008

17. Vanderwal T, Kelly C, Eilbott J, Mayes LC, Castellanos FX. Inscapes: A movie paradigm to improve compliance in functional magnetic resonance imaging. *Neuroimage*. 2015;122:222-232. doi:10.1016/j.neuroimage.2015.07.069

18. Esteban O, Markiewicz CJ, Blair RW, et al. fMRIPrep: a robust preprocessing pipeline for functional MRI. *Nat Methods*. 2019;16(1):111-116. doi:10.1038/s41592-018-0235-4

19. Esteban O, Markiewicz CJ, Goncalves M, DuPre, Elizabeth Kent, James D. Salo, Taylor Ciric, Rastko Pinsard, Basile Blair, Ross W. Poldrack, Russell A. Gorgolewski KJ. fMRIPrep: a robust preprocessing pipeline for functional MRI. Published online 2020. doi:10.5281/zenodo.4252786

20. Gorgolewski K, Burns CD, Madison C, et al. Nipype: A flexible, lightweight and extensible neuroimaging data processing framework in Python. *Front Neuroinform*. 2011;5. doi:10.3389/fninf.2011.00013

21. Gorgolewski KJ, Esteban O, Ellis DG, et al. Nipype: a flexible, lightweight and extensible neuroimaging data processing framework in Python. 0.13.1. Published online May 21, 2017. doi:10.5281/ZENODO.581704

22. Tustison NJ, Avants BB, Cook PA, et al. N4ITK: Improved N3 bias correction. *IEEE Trans Med Imaging*. 2010;29(6):1310-1320. doi:10.1109/TMI.2010.2046908

23. Dale A, Fischl B, Sereno MI. Cortical Surface-Based Analysis: I. Segmentation and Surface Reconstruction. *Neuroimage*. 1999;9(2):179-194. doi:10.1006/nimg.1998.0395

24. Klein A, Ghosh SS, Bao FS, et al. Mindboggling morphometry of human brains. *PLoS Comput Biol*. 2017;13(2). doi:10.1371/journal.pcbi.1005350

25. Avants BB, Epstein CL, Grossman M, Gee JC. Symmetric diffeomorphic image registration with cross-correlation: Evaluating automated labeling of elderly and neurodegenerative brain. *Med Image Anal*. 2008;12(1):26-41. doi:10.1016/j.media.2007.06.004

© 2023 Vandewouw MM et al. *JAMA Network Open.*

26. Fonov V, Evans AC, Botteron K, Almli CR, McKinstry RC, Collins DL. Unbiased average age-appropriate atlases for pediatric studies. *Neuroimage*. 2011;54(1):313-327. doi:10.1016/j.neuroimage.2010.07.033

27. Fonov V, Evans A, McKinstry R, Almli C, Collins D. Unbiased nonlinear average age-appropriate brain templates from birth to adulthood. *Neuroimage*. 2009;47:S102. doi:10.1016/s1053-8119(09)70884-5

28. Zhang Y, Brady M, Smith S. Segmentation of brain MR images through a hidden Markov random field model and the expectation-maximization algorithm. *IEEE Trans Med Imaging*. 2001;20(1):45-57. doi:10.1109/42.906424

29. Cox RW. AFNI: Software for analysis and visualization of functional magnetic resonance neuroimages. *Computers and Biomedical Research*. 1996;29:162-173. doi:10.1006/cbmr.1996.0014

30. Jenkinson M, Bannister P, Brady M, Smith S. Improved optimisation for the robust and accurate linear registration and motion correction of brain images. *Neuroimage*. 2002;17(2):825–841. doi:10.1016/S1053-8119(02)91132-8

31. Huntenburg JM, Gorgolewski KJ, Anwander A, Margulies DS. Evaluating nonlinear coregistration of BOLD EPI and T1 images. In: *20th Annual Meeting of the Organization for Human Brain Mapping*. ; 2014:1.

32. Wang S, Peterson DJ, Gatenby JC, Li W, Grabowski TJ, Madhyastha TM. Evaluation of field map and nonlinear registration methods for correction of susceptibility artifacts in diffusion MRI. *Front Neuroinform*. 2017;11. doi:10.3389/fninf.2017.00017

33. Treiber JM, White NS, Steed TC, et al. Characterization and correction of geometric distortions in 814 Diffusion Weighted Images. *PLoS One*. 2016;11(3). doi:10.1371/journal.pone.0152472

34. Greve DN, Fischl B. Accurate and robust brain image alignment using boundary-based registration. *Neuroimage*. 2009;48(1):63-72. doi:10.1016/j.neuroimage.2009.06.060

35. Power JD, Mitra A, Laumann TO, Snyder AZ, Schlaggar BL, Petersen SE. Methods to detect, characterize, and remove motion artifact in resting state fMRI. *Neuroimage*. Published online 2014. doi:10.1016/j.neuroimage.2013.08.048

36. Lindquist MA, Geuter S, Wager TD, Caffo BS. Modular preprocessing pipelines can reintroduce artifacts into fMRI data. *Hum Brain Mapp*. 2019;40(8):2358-2376. doi:10.1002/hbm.24528

37. Rosenbaum PR, Rubin DB. Constructing a control group using multivariate matched sampling methods that incorporate the propensity score. *American Statistician*. 1985;39(1):33-38. doi:10.1080/00031305.1985.10479383

© 2023 Vandewouw MM et al. *JAMA Network Open.*

38. Johnson WE, Li C, Rabinovic A. Adjusting batch effects in microarray expression data using empirical Bayes methods. *Biostatistics*. 2007;8(1):118-127. doi:10.1093/biostatistics/kxj037

39. Hunt BAE, Wong SM, Vandewouw MM, Brookes MJ, Dunkley BT, Taylor MJ. Spatial and spectral trajectories in typical neurodevelopment from childhood to middle age. *Network Neuroscience*. 2019;3(2):497-520. doi:10.1162/netn_a_00077

40. Wang L, Su L, Shen H, Hu D. Decoding Lifespan Changes of the Human Brain Using Resting-State Functional Connectivity MRI. *PLoS One*. 2012;7(8). doi:10.1371/journal.pone.0044530

41. Da-ano R, Masson I, Lucia F, et al. Performance comparison of modified ComBat for harmonization of radiomic features for multicenter studies. *Sci Rep*. 2020;10(1). doi:10.1038/s41598-020-66110-w

42. Efron B. Bootstrap methods: another look at the jackknife. *Ann Stat*. 1979;7(1):1-26.

43. Johnson WE, Li C, Rabinovic A. Adjusting batch effects in microarray expression data using empirical Bayes methods. *Biostatistics*. 2007;8(1):118-127. doi:10.1093/biostatistics/kxj037

44. Markello RD, Shafiei G, Tremblay C, Postuma RB, Dagher A, Misic B. Multimodal phenotypic axes of Parkinson’s disease. *NPJ Parkinsons Dis*. 2021;7(1). doi:10.1038/s41531-020-00144-9

© 2023 Vandewouw MM et al. *JAMA Network Open.*
