# Supplementary material for: Identifying Replicable Subgroups in Neurodevelopmental Conditions Using Resting-State Functional Magnetic Resonance Imaging Data
Source: JAMA Netw Open. 2023 Mar 13;6(3):e232066. doi: 10.1001/jamanetworkopen.2023.2066 (PMC10011941; doi:10.1001/jamanetworkopen.2023.2066)
Supplement: Supplement 2. — Data Sharing Statement [file jamanetwopen-e232066-s002.pdf]

Data Sharing Statement

Vandewouw. Identifying Replicable Subgroups in Neurodevelopmental Conditions Using Resting-State Functional Magnetic Resonance Imaging Data. *JAMA Netw Open*. Published March 13, 2023. doi:10.1001/jamanetworkopen.2023.2066

Data
Data available: Yes
Data types: Deidentified participant data
How to access data: Participants were drawn from the Province of Ontario Neurodevelopmental Disorders (POND) network (exported April 2021; now available via a controlled data release through Ontario Brain Institute's Brain-CODE: https://www.braincode.ca/) and the Healthy Brain Network (exported November 2020; http://fcon_1000.projects.nitrc.org/indi/cmi_healthy_brain_network/) datasets.
When available: beginning date: 04-01-2021

Supporting Documents
Document types: None

Additional Information
Who can access the data: N/A
Types of analyses: N/A
Mechanisms of data availability: N/A
